# Determination of Folate Bioavailability From Brewer's Yeast in a Dual Isotope Randomized Human Intervention Study

**DOI:** 10.1002/mnfr.70453

**Published:** 2026-04-09

**Authors:** Nadine Weber, Lisa Striegel, Yvonne Methner, Beate Brandl, Viola Groehn, Jean‐Pierre Knapp, Thomas Skurk, Michael Rychlik

**Affiliations:** ^1^ Chair of Analytical Food Chemistry Technical University of Munich Freising Germany; ^2^ Research Center Weihenstephan for Brewing and Food Quality Technical University of Munich Freising Germany; ^3^ ZIEL, Institute for Food & Health, Core Facility Human Studies Technical University of Munich Freising Germany; ^4^ Merck & Cie KmG Schaffhausen Switzerland; ^5^ Technical University of Munich, School of Medicine and Health Klinikum Rechts Der Isar Munich Germany; ^6^ Centre For Nutrition and Food Sciences University of Queensland Brisbane Australia

**Keywords:** bioavailability, dual isotope design, human study, intrinsic labelling, stable isotope dilution assay, yeast folates

## Abstract

An adequate intake of folates is primarily dependent on the amount ingested, but also on the bioavailability of these water‐soluble vitamins belonging to the vitamin B9 group. Therefore, we here present a human pilot study with a novel dual isotope design to assess the bioavailability of brewer's yeast folate as a model food. The particular advantage of the study design is the use of yeast intrinsically labeled with N‐15, and a new reference folate specifically labeled with C‐13. In this trial, 6 volunteers consumed (1) the intrinsically ^15^N‐labeled yeast, (2) a ^13^C_6_‐labeled (6S)‐5‐methyltetrahydrofolate as the reference dose and (3) a folate‐free food on one of the three study days. On each study day, 21 plasma samples were taken and analyzed for three different isotopologues of (6S)‐5‐methyltetrahydrofolate using a ^13^C_5_‐labeled (6S)‐5‐methyltetrahydrofolate as quantitation standard in stable isotope dilution assays. The main outcome of the study was that the yeast folates revealed for the five assessed volunteers a mean bioavailability of 74 % relative to 5‐methyltetrahydrofolate as the reference dose. The study allows to conclude that intrinsically labeled foods are best suited to accurately measure the bioavailabilities of natural food folates embedded in their natural food matrix.

## Graphical abstract

A human pilot study with a novel dual isotope design was performed to assess the bioavailability of brewer's yeast folate as a model food. The study‘s outcome was that the folates from intrinsically ^15^N‐labeled yeast revealed a mean bioavailability of 74 %.

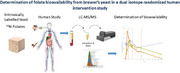

**Abbreviations**
10‐formyl‐folate: 10‐CHO‐PteGlu
5‐CH_3_‐H_4_PteGlu: 5‐methyl‐tetrahydrofolate
5‐CHO‐H_4_PteGlu: 5‐formyl‐tetrahydrofolate
A: analyte
ACN: acetonitrile
AUC: area under the curve
BIA: bioimpedance measurement
BMI: body mass index
DTT: dithiothreitol
H_4_PteGlu: 5,6,7,8‐tetrahydrofolate
HPLC/DAD: high performance liquid chromatography‐diode array detection
IS: internal standard
LC‐MS/MS: liquid chromatography‐tandem mass spectrometry
LoD: Limits of Detection
LoQ: Limit of Quantification
MeOH: methanol
MES: 2‐(N‐morpholino)ethanesulfonic acid)
MRM: multiple reaction monitoring
MTHFR: 5,10‐methylenetetrahydrofolate reductase
NTD: neural tube defect
PteGlu: folic acid
RDI: recommended daily intake
SAX: strong anion‐exchange
SNP: single nucleotide polymorphism
SPE: solid‐phase extraction

## 1 Introduction

The group of folates, also known as vitamin B9, are vital micronutrients. These water‐soluble vitamins are essential for cell growth and renewal and serve as coenzymes in the metabolism of one‐carbon groups and DNA synthesis. The different forms of folate vary chemically in their oxidation state and substituents such as methyl or formyl groups [1]. Since humans cannot synthesize folates internally, they must be ingested through diet or nutritional supplements [2].

Ensuring the recommended daily intake (RDI) of folates is particularly important for pregnant women and women of childbearing age (RDI of 600 µg), as a deficiency is correlated with the risk of neural tube defects (NTDs) and other fetal malformations. Moreover, low folate levels have been associated with higher risks of cardiovascular diseases and colorectal cancer, depression [3, 4]. Particularly in Europe, a tendency towards suboptimal supply has been observed to be correlated particularly with the risk for NTDs [5].

To overcome this undersupply, fortification programs for cereals and cereal products have been implemented in several countries, along with awareness campaigns about folates [6, 7, 8, 9]. However, given the adverse effects of excessive folic acid intake (over 1 mg/day), such as masking vitamin B12 deficiency, accumulation of unmetabolized folic acid, and potential increased cancer risk [10], many researchers are exploring alternative sources of natural folates that are more stable, safer, and more effective [11].

One approach is to identify new natural sources of folate, such as fruits and vegetables, either unknown for their folate content or underutilized [12]. Moreover, other known sources such as yeasts or microalgae may be used as supplements or food ingredients. Another option is biofortification, which involves enhancing the natural folate content of foods, for example, through specific folate‐producing microorganisms like lactic acid bacteria [13] or yeasts [14], or breeding and genetic engineering [15].

Unfortunately, a high folate content in food does not always ensure adequate supply for humans, as the bioavailability of folates can vary significantly. Bioavailability, in this context, refers to the percentage of ingested folates that are absorbed by the organism and available for metabolic processes or storage [16]. The overall mean bioavailability of folates from various food sources is estimated to be 50% relative to a folic acid supplement, influenced by several factors that may inhibit folate absorption and metabolism [17].

Food‐related factors such as the distribution of polyglutamate forms and folate C1‐vitamers, the food matrix composition, dietary fiber, low pH levels, and the instability of labile folate vitamers in food and during digestion can all hinder folate absorption and, therefore, its bioavailability. Conversely, specific stabilizing food components like folate‐binding proteins, ascorbate, and zinc can enhance folate stability and absorption [18].

Individual factors impacting bioavailability may include 5,10‐methylenetetrahydrofolate reductase (MTHFR) gene polymorphisms, as well as sex and ethnicity. MTHFR polymorphisms can affect the 5‐CH_3_‐H_4_PteGlu response to food folate if the latter consists of other folate vitamers. Therefore, the conversion of food folates via H_4_PteGlu and 5,10‐methylenetetrahydrofolate to 5‐CH_3_‐H_4_Pte by MTHFR may be reduced and thus impair apparent bioavailability [19, 20, 21]. Understanding to which extent nutrients are absorbed and made bioavailable in conjunction with dietary components can help individuals ensure adequate nutrient intake. These insights can also aid in designing foods, meals, and diets that effectively deliver bioavailable nutrients to specific target populations. Human studies are essential for understanding the bioavailability of folates from various food sources and generating the data necessary for comprehensive dietary recommendations. With regard to folate bioavailability, human studies can be performed as long‐term intervention studies with monitoring long‐term status markers or short‐term studies measuring plasma 5‐methyltetrahydrofolate (5‐CH_3_‐H_4_PteGlu) levels and calculating bioavailability from area under the curves (AUC) data in relation to the dosage of a reference folate, for which a high bioavailability is assumed [22]. Whereas initially folic acid (PteGlu) has been used as a reference due to its stability [23], for assessing the folate bioavailability from strawberries [24] we recently used 5‐CH_3_‐H_4_PteGlu as the reference, which revealed superior properties such as a more similar plasma kinetic to natural folates. Additionally, the EFSA Panel on Dietetic Products, Nutrition and Allergies stated “ the bioavailability of supplemental L‐5‐methyl‐THF (calcium salt of (6S)‐5‐methyl‐tetrahydrofolic acid or calcium‐L‐methyl‐PteGlu) has been reported to be similar to that of folic acid at equimolar doses of supplemental folic acid between 100 and 400 µg/day” [25]. However, the absolute bioavailability of 5‐CH_3_‐H_4_PteGlu has been controversially discussed as an oral dosage versus an i.m. injection with two volunteers showed a much higher plasma appearance of the i.m. dose [26]. This result, however, can be questioned as a possible bias due to fluctuations of the individual basal predose 5‐methyltetrahydrofolate concentrations cannot be ruled out. Additionally, a potential partial hepatic retention was also supposed by the same authors [22], which might have reduced appearance in comparison to the i.m. dose although absorption may have been higher.

Of all food components and ingredients, baker's or top fermenting brewer's yeast presents itself as one of the best folate sources with contents far above 4000 µg/100 g [27]. Moreover, its potential to contribute to vitamin and protein supply for humans in future biotechnological approaches like Power‐to‐Protein or Power‐to‐Vitamin as an alternative to traditional foods based on animals or legumes has been highlighted [28]. In the past, yeast folate bioavailability has been rarely investigated and only one long‐term intervention study revealed a bioavailability of 59 % relative to folic acid [29]. However, the latter study design has been questioned and for accurate recommendations whether to include more yeast in the general diet, further evidence is needed. Therefore, we here present a short‐term human pilot study to determine the folate bioavailability of yeast.

As we learned from our previous experience with short‐ term intervention studies [23, 30, 31] AUC calculations from plasma folate levels often bear the problem that base folate levels are not zero, and therefore, superposed AUCs cannot be measured accurately as baseline levels cannot be assumed to be constant [23].

Therefore, the idea was to use stable isotope‐labeled folates to circumvent this problem, and, unprecedentedly to use labeled isotopologues both for the reference folate and for yeast folates. The labelling of yeast folates appeared very straightforward by cultivating yeast in a synthetic medium containing labeled ^15^N as the only nitrogen source. In this way, the yeast folates would be intrinsically labeled, which is a further advantage over test foods in previous bioavailability studies, where synthetically labeled folates have been added. Therefore, intrinsically labeled foods reflect the real inclusion of the folates in the surrounding matrix and the accessibility of the folates during digestion. For yeast, we could already show that folates are trapped in the cells and only released when the cells are disrupted [32]. In this way, we used, on the one hand, one labeled folate as the reference and, on the other hand, differently labeled intrinsic folates in yeast to calculate unambiguously the different AUC levels. Quantitation of all folate isotopologues was performed by liquid chromatography‐tandem mass spectrometry (LC‐MS/MS).

## 2 Materials & Methods

### 2.1 Chemicals

The HPLC‐ and LC‐MS grade solvents acetonitrile (ACN), methanol (MeOH), and water were purchased from VWR (Ismaning, Germany). Ascorbic acid, formic acid (> 95%), and 2‐(N‐morpholino)‐ethanesulfonic acid (MES) were obtained from Sigma‐Aldrich (Steinheim, Germany). Potassium dihydrogen phosphate, sodium acetate trihydrate, and sodium hydroxide were purchased from Merck (Darmstadt, Germany), disodium hydrogen phosphate (anhydrous) and sodium chloride were obtained from Alfa Aesar and Baker J.T. (Thermo Fisher, Karlsruhe, Germany). Rat serum and chicken pancreas containing γ‐glutamyl hydrolase (EC 3.4.19.9) were purchased from Biozol in Eching, Germany, and Difco in Sparks, MD, USA, respectively, and dithiothreitol (DTT) was obtained from Sigma‐Aldrich. The unlabeled reference compounds ((6*S*)‐H_4_PteGlu, (6*R,S*)‐5‐CH_3_‐H_4_PteGlu, (6 *R,S*)‐5‐CHO‐H_4_PteGlu, PteGlu, and 10‐CHO‐PteGlu) were purchased from Schircks Laboratories in Jona, Switzerland. The isotopological internal standards (IS) (Pte[^13^C_5_]Glu, (6*S*)‐H_4_Pte[^13^C_5_]Glu, (6*S*)‐5‐CH_3_‐H_4_Pte[^13^C_5_]Glu‐Ca, (6*S*)‐5‐CHO‐H_4_Pte[^13^C_5_]Glu‐Ca, and 10‐CHO‐Pte[^13^C_5_]Glu) were obtained from Merck & Cie KmG in Schaffhausen, Switzerland. The reference compound [^13^C‐Ph]_6_‐(6S)‐5‐CH_3_‐H_4_PteGlu was a gift from Merck & Cie KmG. The Strata strong anion exchange (SAX) cartridges (quaternary amine, 100 mg/1 mL and 500 mg/3 mL) for solid‐phase extraction were from Phenomenex in Aschaffenburg, Germany.

### 2.2 Buffers & Solutions

The methods described by Striegel et al. [24] for folate quantification in blood plasma and Obermaier et al. [12] for folate analysis in food were followed for the preparation of buffers, stock solutions, enzymes, and extraction processes with slight modifications.

For the extraction buffer 2 g/L of ascorbic acid and MES (200 mmol/L) were dissolved in a solution containing 0.1 g/L of DTT and adjusted to pH 5 using aqueous NaOH (5 mol/L). Phosphate buffer (100 mmol/L) for dissolving folate standards and as part of the equilibration buffer for SAX cartridges was obtained by adjusting a 100 mmol/L disodium hydrogen phosphate aqueous solution to pH 7.0 using a potassium dihydrogen phosphate (100 mmol/L) aqueous solution. The equilibration buffer for SAX cartridges was prepared by mixing 0.2 g/L of DTT and 10 mmol/L phosphate buffer with deionized water. The two different elution buffers consisted of 50 g/L (for monoglutamate analysis) respectively, 100 g/L aqueous sodium chloride (for polyglutamate analysis), 100 mmol/L aqueous sodium acetate, 0.1 g/L of DTT, and 10 g/L of ascorbic acid.

For enzymatic deconjugation, lyophilized chicken pancreas (30 mg) was added to a 30 mL aqueous phosphate buffer solution (100 mmol/L) containing 1% ascorbic acid, adjusted to pH 7. Both the chicken pancreas enzyme and the enzyme present in rat serum were treated with activated carbon for 30 min and filtered through a 0.45 µm molecular filter.

To prepare the stock solutions of the reference compounds, 10 mg of PteGlu and 2 mg each of H_4_PteGlu, 5‐CH_3_‐H_4_PteGlu, 5‐CHO‐H_4_PteGlu, and 10‐CHO‐PteGlu were dissolved in 3 and 10 mL (for PteGlu) phosphate buffer, respectively, and brought up to 10 mL and 100 mL (for PteGlu) using the extraction buffer. The exact concentrations of the freshly prepared unlabeled analytes were determined on each extraction day using high performance liquid chromatography‐diode array detection (HPLC/DAD) with PteGlu as the IS for 5‐CH_3_‐H_4_PteGlu, 5‐CHO‐H_4_PteGlu, and 10‐CHO‐PteGlu, and 5‐CH_3_‐H_4_PteGlu as the IS for the determination of H_4_PteGlu as detailed below. For LC‐MS/MS analysis, the stock solutions were further diluted at 1:20 and 1:10 (for PteGlu). The labeled standards ([^13^C_5_]‐PteGlu, [^13^C_5_]‐H_4_PteGlu, [^13^C_5_]‐5‐CH_3_‐H_4_PteGlu, [^13^C_5_]‐5‐CHO‐H_4_PteGlu, and [^13^C_5_]‐10‐CHO‐PteGlu) were dissolved to concentrations of 80 ‐ 120 µg/mL in extraction buffer and further diluted to a final concentration of 8 ‐ 11 µg/mL. The labeled reference solutions were stored in the dark at −20°C.

For washing the yeast, a phosphate‐buffered saline (PBS) buffer was prepared. For this, sodium chloride (137 mmol/L), potassium chloride (2.68 mmol/L), disodium hydrogenphosphate (9.93 mmol/L), and potassium dihydrogenphosphate (1.98 mmol/L) were dissolved in distilled water.

### 2.3 Yeast Preparation

For preparing the synthetic culture media for yeast cultivation, first the components from Table S1 part A were dissolved in 2L distilled water and autoclaved (20 min, 121°C). Then, the vitamins listed in Table S1 part B were dissolved in 6 mL distilled water and sterile filtered in the media from part A.

After inoculating the media with the top‐fermenting *Saccharomyces cerevisiae* strain (LeoBavaricus‐TUM 68, (BLQ, Weihenstephan, Germany), incubation at room temperature on a shaker was performed in a culture flask while shaking for 5 days. To improve the yield, the yeast was inoculated in a small 100 mL culture flask with 40 mL of the prepared medium for 2 days and was transferred to the 2L culture flask for further 3 days. 1 mL of the vitamin mix (Part B) was added to the 100 mL flask and the rest was added to the 2 L flask after 2 days. The vitamin mix was stored in the fridge (+4°C) up to use. Thereafter, the resulting suspension was centrifuged, the residue washed three times with PBS buffer, again centrifuged and the obtained yeast was lyophilized. Analysis of folates was performed as detailed below.

After choosing a suitable yeast strain the folate production was improved by comparing different variations of the medium composition. Six different media were tested, each based on the basic medium shown in Table S1. In Table 1 only the adjusted variable is shown. The preparation for all media was performed as described above. For medium C‐F the compounds of Table S1 Part A were dissolved in phosphate buffer instead of distilled water and adjusted to different pH.

**TABLE 1:** Variables to the basic medium composition for improving the folate production of the yeast.

Medium	Variable	Concentration of the respective variable
A	Ammonium chloride	40 g/L
B	+ Ergosterol	100 µl/L
C	Phosphate buffer, pH 5.5	50 mmol/L
D	Phosphate buffer, pH 5.5	100 mmol/L
E	Phosphate buffer, pH 6.0	50 mmol/L
F	Phosphate buffer, pH 6.5	50 mmol/L

### 2.4 Test Foods

The following intervention items were applied during the human study:

1. Capsules containing the “Test food”: The lyophilized ^15^N‐labeled yeast (8 g, equivalent to 250 mg per capsule,) prepared as described before was filled in 32 capsules (white VCAPS^TM,^ size 1, Kapselwelt, Hude, Germany) as test food for each study subject.
2. Capsules containing the “Reference folate”: The compound [^13^C‐Ph]_6_‐5‐CH_3_‐H_4_PteGlu (400 µg) was filled in one capsule and further 16 capsules were filled with cellulose (approx. 2 g in total, equivalent to 125 mg per capsule, microcrystalline, Ph.Eur., OTC Pharma GmbH, Bönen, Germany) complemented the reference dose for each volunteer.
3. Capsules containing the “Folate‐free” test food: Cellulose (approx. 2 g in total, equivalent to 125 mg per capsule) was filled in 17 capsules and served as the control.

The night before the study days the subjects ate a standardized meal containing rice, butter, and salt. During the study days the study subjects were allowed to eat standardized foods low in folates: honey, rice, and corn wafers, and Edam cheese and Gouda. (The analyzed folate contents of the different food items are indicated in Table S2.) In a pre‐study with one study subject, the latter food items were considered suitable for use as standardized meals before and during the study because no increase in the plasma folate concentration was observed after consumption in the pre‐trial.

### 2.5 Folate Analysis of Food

The standardized foods for the human study were subjected to a triple analysis, following the protocol outlined by Striegel et al. [24] and Obermaier et al. [12]. In contrast to the plasma samples, we evaluated the five most important folate monoglutamate vitamers to cover the total folate content, namely PteGlu, H_4_PteGlu, 5‐CH_3_‐H_4_PteGlu, 5‐CHO‐H_4_PteGlu, and 10‐CHO‐PteGlu.

For quantitation of total folate in foods, 5–100 mg of freeze‐dried samples were used. After a 15‐min equilibration with 10 mL extraction buffer, the ISs were added in quantities equivalent to the expected concentration of the analytes in the sample. Following another 15‐min equilibration time and a subsequent 10‐min boiling step, a mixture of 900 µL chicken pancreas solution and 400 µL rat serum was used for deconjugation. The samples were then incubated overnight at 37°C, followed by a 10‐min boiling step and adding 10 mL acetonitrile to the cooled samples. The resulting supernatant from each sample was purified with a solid‐phase extraction (SPE) using SAX cartridges (3 mL, 500 mg). After membrane filtration, the purified samples were subjected to liquid chromatography‐tandem mass spectrometry (LC‐MS/MS). Besides the samples, a response mixture for all analytes and ISs and the blank was measured. For the blank 10 mL equilibration buffer were spiked with 5 µL of each labeled standard and prepared like the samples. The evaluation of the endogenous number of folates in each sample was done by subtracting the folate content of the chicken pancreas and the rat serum determined in the blank measurement. All presented outcomes are normalized based on dry biomass.

Quantitation of unlabeled folates by generating the respective response curves was performed as outlined by Obermaier et al. [12]. For quantitation of ^15^N‐labeled folates in intrinsically labeled yeast sample workup was done analogously to the other foods detailed above. In contrast to the quantitation of the unlabeled folates, in LC‐MS/MS the respective multiple reaction monitoring (MRM) traces for the ^15^N_7_‐labeled monoglutamates were monitored and the contents were calculated by using the MRMs for ^13^C_5_‐labeled ISsand the response curves obtained for quantitation of the unlabeled folates.

The polyglutamate forms of folates were analyzed as reported previously [26]. In brief, quantification of 5‐CH_3_‐H_4_PteGlu_1‐7_ was achieved by omitting the deconjugation step and using ^13^C_5_‐5‐CH_3_‐H_4_PteGlu as the IS. SPE purification on SAX was performed as outlined in [26] and LC‐MS/MS quantitation was achieved by using matrix‐matched calibration with the instrumental conditions outlined below.

### 2.6 Plasma Sampling and Analysis

The sampling and analysis of the blood plasma was done according to Obermaier et al. [30]. After sample collection, venous blood was centrifuged at 4°C for 10 min, and the plasma was transferred to an Eppendorf tube containing 20 mg of ascorbic acid. The plasma samples were stored in the dark at −20°C until further processing.

To quantify the plasma folate content, 60 µL plasma was mixed with 1 mL of extraction buffer, and the corresponding amount of IS [^13^C_5_]‐5‐CH_3_‐H_4_PteGlu was added. After 30 min of equilibration at room temperature with stirring, 1 mL ACN was added, and the mixture was centrifuged for 20 min at 4°C. The plasma mix was applied to SPE with SAX cartridges (1 mL, 100 mg). The eluate was membrane‐filtered and stored in the dark at −20°C until measurement by LC‐MS/MS.

All vitamers were analyzed in our LC‐MS/MS method, but only 5‐CH_3_‐H_4_PteGlu was evaluated as this vitamer was the only detectable folate form in blood plasma. The assessment of relative bioavailability was conducted with reference to [^13^C‐Ph]_6_‐5‐CH_3_‐H_4_PteGlu as the reference compound, which is assumed to have a high absorption rate (c.f. discussion in the introduction).

### 2.7 Method Validation of the Plasma Assay

#### 2.7.1 Calibration and Quantification

For the response curve 13 calibration points with constant amounts of IS with varying amounts of analyte (A) were measured. The molar ratios [n(A)/n(IS)] were between 0.01 and 15. Linear regression analysis was performed by combining the molar ratios [n(A)/n(IS)] with the peak areas [A(A)/A(IS)] from the LC‐MS/MS measurements. To confirm the linearity, Mandel´s test was conducted [33]. For quantitation of [^15^N_7_]‐5‐CH_3_‐H_4_PteGlu the respective MRMs were monitored and the contents were calculated by using the MRMs for the ^13^C_5_‐labeled IS and the calibration curves obtained for quantitation of unlabeled 5‐CH_3_‐H_4_PteGlu.

#### 2.7.2 Limit of Detection and Quantification

The limits of detection and quantification (LoD, LoQ) were determined according to Vogelgesang and Hädrich [34], where the LoD is “three times as high as the largest possible signal from a blank analysis”. Moreover, the LoQ is ten times higher than the value obtained from blank analysis. In this study, the blank matrix was a recombinant consisting of water (98.6%), NaCl (0.91%), freeze dried egg white (0.07%) and sunflower oil (0.06%) in order to represent blood plasma. For the determination of the LoD and LoQ the matrix was spiked with the analytes (^13^C‐[Ph]_6_ 5‐CH_3_‐H_4_PteGlu and 5‐CH_3_‐H_4_PteGlu) at four different amounts. The extraction work‐up was done in triplicate for each level. The lowest concentration was slightly above the estimated LoD and the highest concentration tenfold higher. At each level, the concentrations of the labeled standards were equal to 0.01 nmol/L. The correlation of the IS and the analytes provided a new, expanded calibration function. From the correlation analysis prediction intervals were obtained, which were used to determine the LoD and LoQ [34].

#### 2.7.3 Precision

To evaluate the precision of the measurement regarding the labeled analyte; intra‐day, inter‐day and inter‐injection precision measurements were performed similarly as in our previous study [12]. Therefore, two of the analyzed plasma samples were used, one for each analyte. The inter‐injection precision was determined with one sample (*n* = 1) injected 15 times in a row (*i* = 15). For determining the inter‐day precisions one sample was analyzed in triplicate within three independent extractions (*n* = 9) and triplicate injections (*i* = 27) over three weeks. The intra‐day precision was calculated with one sample in a triplicate extraction (*n* = 3) and triplicate measurement (*i* = 9).

#### 2.7.4 Recovery

Blank matrix was spiked in triplicate with the analytes in three different concentrations (1.3 nmol/L, 16.3 nmol/L and 81.3 nmol/L) and the normal work‐up was conducted. The recoveries were calculated as the ratio of the detected and spiked contents, whereas the values should be between 70% and 120% according to Vogelgesang and Hädrich [34].

#### 2.7.5 Instrumental Conditions

The instrumental conditions for the analysis were based on Striegel et al. [35] and Obermaier et al. [31].

To check the purity and quantifying the actual concentrations of the unlabeled reference solutions, a Shimadzu HPLC/DAD system (Shimadzu, Kyoto, Japan) was used with a reversed‐phase column (C18 EC, 250×3 mm, 5 µm, 100 Å, precolumn: C18, 8×3 mm, Machery‐Nagel, Düren, Germany). Therefore, PteGlu was used as the IS for 5‐CH_3_‐H_4_PteGlu, 5‐CHO‐H_4_PteGlu, and 10‐CHO‐PteGlu and 5‐CH_3_‐H_4_PteGlu is used as the IS for the determination of H_4_PteGlu. A sample volume of 10 µL of the pure folate solution or a mixture of the folate and the respective IS was injected, and the column oven temperature was set to 25°C. The mobile phases consisted of (A) 0.1% acetic acid and (B) methanol. The analysis was performed at a flow rate of 0.4 mL/min. The gradient started at 10% B, followed by a 7‐min equilibration time. Subsequently, B concentration was linearly increased to 50% within the next 14 min. The gradient then linearly reached 100% B in 2 min and was held at 100% B for 1 min. Finally, the mobile phase returned to the starting condition (10% B) in 2 min and was equilibrated for 9 min before the next run.

For the LC‐MS/MS measurement, a Shimadzu Nexera X2 UHPLC system (Shimadzu, Kyoto, Japan) with a Raptor ARC‐18 column (2.7 µm, 100×2.1 mm, Restek, Bad Homburg, Germany) and a Raptor ARC‐18 precolumn (2.7 µm, 5×2.1 mm, Restek, Bad Homburg, Germany) as the stationary phase was used for the plasma and food method. The injection volume was −10 µL, and the column temperature was 30°C. For the polyglutamate analysis a YMC‐Pack Pro C18 RP (150×3.0 mm, 3 µm, YMC, Kyoto, Japan) column with a C18 pre column (4×2.0 mm, Phenomenex, Aschaffenburg, Germany) was used. The injection volume for this method was 20 µL and the column temperature was 30°C.

The mobile phases consisted of (A) 0.1% formic acid and (B) ACN with 0.1% formic acid. The analysis was conducted at a flow rate of 0.4 mL/min. The gradient elution for plasma sample analysis started at 3% B held for 1 min, followed by a linear increase to 10% B within the next 2 min, which was then held for 2.5 min. Subsequently, the gradient linearly increased to 15% B over 5 min and to 50% B within 1 min, where it was maintained for 1 min. Finally, the gradient returned to the starting condition (3% B) within 4 min.

For the polyglutamate analysis of yeast folates, the gradient elution started at 5% B and held for 2 min, followed by a linear increase to 10% B within the next 6 min, which was then held for 3 min. Subsequently, the gradient linearly increased to 15% B over 4 min and to 90% B within 2 min, where it was maintained for 2 min. Finally, the gradient returned to the starting condition (5% B) within 2 min.

The triple quadrupole mass spectrometer (LCMS‐8050, Shimadzu, Kyoto, Japan) was operated in the positive ESI mode for all analytes. For optimizing the ion source parameters each labeled and unlabeled standard solution (1 mg/L) was injected. The heat block, desolvation line, and interface temperature were maintained at 400°C, 250°C, and 300°C, respectively. The drying, heating, and nebulizing gas flow were set to 10 L/min, 10 L/min, and 3 L/min, respectively. Collision‐induced dissociation gas was applied at 270 kPa, and the interface voltage was set to 4 kV. The column effluent was only directed to the mass spectrometer from 2.1 to 7.0 min for the plasma and food method and 6.5 to 17.0 min for the polyglutamate analysis. MRM was used for data acquisition. Detailed conditions can be found in the Supplementary materials.

Tables S3 and S4 display the MRM scanning parameters for folate monoglutamate vitamers and their isotopologues in blood plasma and food samples, respectively, and Tables S5 and S6 the MRM scanning parameters for folate polyglutamate vitamers and their isotopologues in yeast.

The measurements were analyzed and evaluated using LabSolutions software 5.109 (Shimadzu, Kyoto, Japan).

#### 2.7.6 Ethical Permission

The ethics application was submitted and approved by the ethics committee of the School of Medicine and Health of the Technical University of Munich (#325/19S). The study was registered in the German Clinical Trial Register (DRKS00022344), and participants provided written informed consent prior to their inclusion into the study.

### 2.8 Human Study

#### 2.8.1 Study Participation

The inclusion criteria for this study were: healthy volunteers, age between 18 and 30 years, both males and females, Caucasians, Body mass index (BMI) < 30 kg/m^2^, nonsmokers, and written informed consent. The study protocol defined the following exclusion criteria: current participation in intervention studies, active smoking, alcohol abuse, vitamin B_12_ deficiency, elevated homocysteine levels, anemia (based on complete blood count including the Hb, erythrocyte volume and staining coefficient), chronic illness, blood donation in the past 3 months, allergy or intolerance to dairy products. The study protocol specified that the following criteria would lead to an exclusion or a drop‐out during the study: antibiotic use during the experimental period, acute illnesses during the test phase, withdrawal of consent to participate, and violation of experimental conditions.

#### 2.8.2 Study Design

**FIGURE 1:**
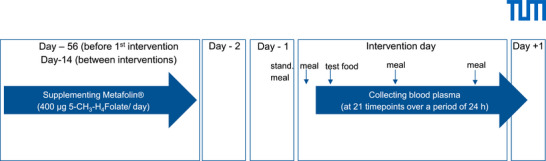
Human study design; stand. meal: standardized meal. For illustration of the cross‐over design please refer to Table S7.

This study was performed between June 2020 and March 2021 in Freising, Germany. A random sample of volunteers from the general population was recruited by advertising from June to July 2020. Aim and procedures were explained during a presentation of the scientific staff at information meetings. The trial was designed as a monocentric randomized controlled intervention study with a crossover design. The interventional study included 3 intervention days (Table S7) with a wash out phase before and during the study days (Figure 1). The primary outcome was the determination of the plasma folate level in blood after the consumption of the labeled folate, the folate supplement and the folate free food. For the secondary outcome the absorption of folates, the difference between the test subjects and the effect of the methylenetetrahydrofolate reductase (MTHFR) genotype was examined. The study was designed as a pilot study, therefore six patients with a 20% drop‐out rate were chosen. For generating pseudonym codes a website (random.org) was used. To ensure blinding and to reduce bias, randomization was conducted via a web‐based system that generated the treatment sequence. Results were placed in sealed, opaque envelopes. The envelopes were numbered consecutively and were opened only after the participant's informed consent was obtained. Both, study physicians and participants were blinded.

Before the start of the study, a comprehensive screening of 19 participants was conducted, including questionnaires on inclusion and exclusion criteria as outlined above, blood tests with the assessment of the homocysteine status, the vitamin B_12_ status, and the hemoglobin status. A genetic analysis was also performed to identify polymorphisms of the methylenetetrahydrofolate reductase gene (MTHFR gene). This gene mutation is known to reduce enzyme activity by 60% [36].

Height, weight, and other anthropometric data were determined using non‐invasive bioimpedance measurements (BIA) for the six study participants. Body composition and weight were measured using the Seca mBCA 515 device (Seca GmbH & Co KG, Hamburg, Germany). For female participants, pregnancy was excluded prior to the study through a conventional pregnancy test (urine test). An overview of the individual subject parameters can be found in Table S8.

Eight weeks before the first study day, participants received a daily dietary supplement as tablets containing 400 µg of (6S)‐5‐CH_3_‐H_4_PteGlu in the form of Metafolin((6S)‐5‐CH_3_‐H_4_PteGlu calcium) to replenish liver and erythrocyte folate stores. During the intervals between the study days, participants first had a 14‐day washout period without supplementation and thereafter continued to take 400 µg of the commercial folate supplement daily for 12 days to maintain folate levels. This supplement was discontinued 2 days before each intervention (wash‐out phase) to deplete and stabilize plasma folate levels. This study design is illustrated in Figure 1.

Participants consumed standardized low‐folate meals consisting of a variability of rice, curry, salt and butter with the opportunity to add the allowed food (Table S2). The evening before each test day, 2 h before the start, and on the test day itself (after 2 and 5 h) the permitted foods, with the exception of rice, were consumed in unlimited quantities. The participants recorded their dietary intake during these meals. On the three test days, venous blood samples (4.9 mL each, totaling 98 mL per test day) were collected from a venous indwelling cannula before consuming the food (0 min) and at 21 subsequent time points (1, 15, 30, 45, 60, 75, 90, 105, 120, 150, 180, 210, 240, 270, 300, 360, 420, 480, 540, 600, and 1440 min). Analyzed food items with a folate content of less than 50 µg/100 g folate were verified for consumption as standardized meals before and during the study (Table S6).

#### 2.8.3 Statistics and Biokinetic Calculations

The analysis was conducted in three extractions (technical triplicates *n* = 3) of each plasma sample and duplicate injections at the LC‐MS/MS instrument. The plasma folate results were calculated as nmol/L, including the relative standard deviation (RSD, %). The results of the analyzed food are expressed in µg/100 g, based on the molecular mass of PteGlu. The error bars in the subsequent figures represent the variation. Statistical tests for significant differences were not performed since the sample data is only indicative and not representative in sample number and size.

For the evaluation of the biokinetics, the following parameters were calculated: c_max_ (maximum plasma concentration), t_max_ (time of c_max_), and the area under the curve (AUC), which is determined by the linear trapezoidal rule via the integral of the concentration‐time curve. To determine the plasma concentration curve, all calculated levels of 5‐CH_3_‐H_4_PteGlu were visualized, and the mentioned parameters were determined. The relative bioavailability was calculated from the dose‐adjusted AUCs in relation to that of the ^13^C‐(Ph_6_)‐5‐CH_3_‐H_4_PteGlu dose.

## 3 Results

### 3.1 Intrinsic Stable Isotope Labelling of Yeast

For intrinsic stable isotope labelling of yeast folates we decided to use ammonium‐^15^N chloride as the only source of nitrogen in a synthetic culture medium, as omitting other nitrogen sources from full media increases the chance of obtaining fully ^15^N‐labeled folates.

In preliminary experiments we aimed at finding a yeast strain capable of producing a high folate content.

Hjortmo et al. [37] investigated the folate content of various yeast strains and found very high folate contents above 4000 µg/100 g, particularly in *Saccharomyces* strains. Based on these results and our previous report [27], a top‐fermenting and very well characterized *Saccharomyces cerevisiae* strain (LeoBavaricus‐TUM 68, was chosen for further experiments).

For the exclusive incorporation of [^15^N] into the yeast folates, the medium needs to provide exclusively labeled nitrogen in a suitable and affordable form. Therefore, we chose one synthetic medium reported in the literature that provides nitrogen from ammonium chloride [38] and designed a further synthetic one with additional elements like sodium chloride, iodine, copper, molybdate, and the vitamin riboflavin. A further modification consisted of the addition of the growth enhancer oleic acid, as the latter has been found to significantly increase the biomass yield [39]. Then, we tried to improve the folate production by optimizing the medium composition and the inoculation process.

There were noticeable differences in the biosynthesis rates of folates between the different media. At first the folate content of medium A, B, and C was compared to the basic medium. The total folate content varies between 9680 ± 180 µg/100 g for the basic medium to 11,550 ± 342 µg/100 g for Medium C. As Medium C showed the highest folate production, it was tested if a variation of the concentration of the phosphate buffer (Medium D) or the pH (Medium E, F) could improve the folate production further. Medium D and Medium E both could improve the folate production by 26%. For the production of test food Medium E was chosen after another cultivation and a slightly higher 5‐CH_3_‐H_4_PteGlu and total folate content.

As intended, replacing unlabeled ammonia chloride with [^15^N]‐ammonia chloride resulted in an essentially complete [^15^N]‐labelling of the yeast folates (Figure S1) with the signal at *m/z* 467 of the [^15^N_7_]‐isotopologue being the only visible one in 5‐CH_3_‐H_4_PteGlu in comparison to the signal of *m/z* 460 of its unlabeled isotopologue. The labeled yeast biomass revealed a total folate content of 7.4 mg/100 g dry mass, which is well in line with our recently reported folate contents in cultivated yeast [27]. To further specify the different vitamers, one has to differentiate between (i) the different C1‐derivatives like 5‐methyl or 10‐formyl derivative or H_4_PteGlu or PteGlu, irrespective of whether they comprise one or several glutamates and (ii) the number of glutamates enclosed irrespective of the C1‐moiety attached. The C1‐derivative distribution revealed 5‐CH_3_‐H_4_PteGlu as the most abundant vitamer (68%) followed by H_4_PteGlu (21 %) and 5‐CHO‐H_4_PteGlu (10%) (Table 2). These data are well in line with our previous report [26], as well as the polyglutamate distribution revealing the heptaglutamate and the hexaglutamate as essentially the only occurring vitamers (Table 3), when considering 5‐CH_3_‐H_4_PteGlu. With these results, the generated yeast appeared well suited for the human study due to its complete labelling and its vitamer distribution consisting mainly of polyglutamate forms.

**TABLE 2:** Vitamer distribution and total folate content of the labeled yeast in [µg 100g^−1^], calculated as PteGlu.

		[^15^N_7_]‐PteGlu	[^15^N_7_]‐H_4_PteGlu	[^15^N_7_]‐5‐CH_3_‐H_4_PteGlu	[^15^N_7_]‐5‐CHO‐H_4_PteGlu	[^15^N_7_]‐10‐CHO‐PteGlu	Total Folate [µg/100 g]
Test Food [^15^N]‐ Folate	ø [µg/100 g] ± SD [µg/100 g]	< LOD^a^	1540 ± 49.2	5050 ± 217	746 ± 74.8	69.9 ± 6.46	7400 ± 276

^a^ Limit of Detection: 0.33 µg/100 g.

**TABLE 3:** Polyglutamate distribution of the labeled yeast in [%], calculated as PteGlu.

	[^15^N_7_]‐5‐CH_3_‐H_4_PteGlu_1_	[^15^N_7_]‐5‐CH_3_‐H_4_PteGlu_2_	[^15^N_7_]‐5‐CH_3_‐H_4_PteGlu_3_	[^15^N_7_]‐5‐CH_3_‐H_4_PteGlu_4_	[^15^N_7_]‐5‐CH_3_‐H_4_PteGlu_5_	[^15^N_7_]‐5‐CH_3_‐H_4_PteGlu_6_	[^15^N_7_]‐5‐CH_3_‐H_4_PteGlu_7_
Test Food [^15^N]‐CH_3_‐H_4_PteGlu_n_ [%]	0.03	0.10	0.20	0.47	0.47	44.5	54.3

### 3.2 LC‐MS/MS Analysis

One methodological challenge of multiple isotope studies is the differentiation of the isotopologues without any crosstalk during mass spectrometry. In the study presented here, the isotologues under study were (i) the major natural isotopolologue 5‐CH_3_‐H_4_PteGlu as the one occurring endogenously in blood plasma, (ii) [^13^C_5_]‐5‐CH_3_‐H_4_PteGlu as our usual isotopologue applied for quantitation of the natural one, (iii) [^13^C‐Ph]_6_‐5‐CH_3_‐H_4_PteGlu as the reference compound applied as the reference for 100% bioavailability and (iv) [^15^N_7_]‐ 5‐CH_3_‐H_4_PteGlu as the respective plasma folate stemming from intrinsically labeled folates in the labeled yeast as the test compound.

**FIGURE 2:**
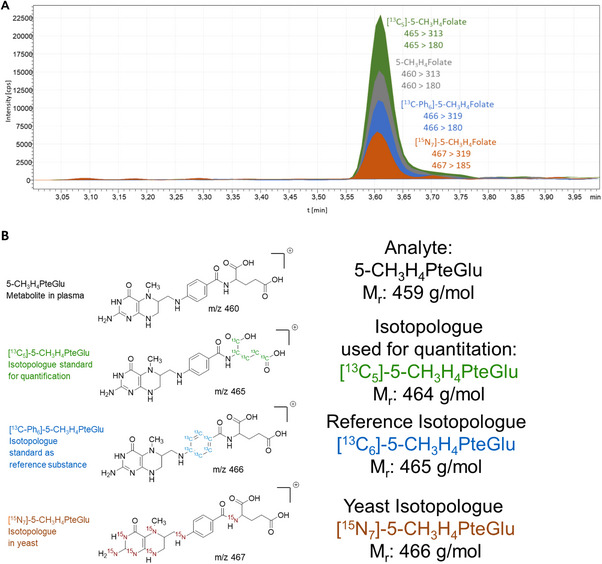
(A) Differentiation of the four isotopologues of 5‐CH_3_‐H_4_PteGlu with their respective qualifier and quantifier transitions in LC‐MS/MS. (B) Structures of the four 5‐CH_3_‐H_4_PteGlu isotopologues (precursor ions).

When comparing the molecular mass ions of the isotopologues, they show, on the one hand, in single stage mass spectrometry a signal at *m/z* 460 for 5‐CH_3_‐H_4_PteGlu and, on the other hand, signals ranging between *m/z* 465 to 467 for the other isotopologues. Given the abundance of naturally occurring isotopologues, the mass difference of 5 Da would be sufficient to differentiate the unlabeled folate from the labeled ones, but not the labeled ones from each other. If all isotopologues must be measured simultaneously, the additional differentiation in tandem mass spectrometry is necessary and possible, as displayed in figure 2. When considering the precursor ion and the signals of the product ions, all four isotopologues can be clearly differentiated. This is due not only to the different number of labeling but also to their labelling position solely in the glutamate part or in the phenyl part or in the whole molecule. As displayed in Figure 2, the differentiation is possible in both shown qualifier and quantifier transitions with the exception of (i) the first transition of [^15^N_7_]‐ 5‐CH_3_‐H_4_PteGlu and [^13^C‐Ph]_6_‐5‐CH_3_‐H_4_PteGlu and of (ii) the second transition of [^13^C‐Ph]_6_‐5‐CH_3_‐H_4_PteGlu and [^13^C_5_]‐5‐CH_3_‐H_4_PteGlu, both showing only the mass increment of 1 Da in the precursor ion and no difference in the production. However, the issue of (i) can be solved when applying the third transition (Table S3), showing a clear mass increment of 5 Da in the production. The possible cross‐talk of the second transition of (ii), however, cannot be resolved with the third transition and, therefore, the quantitation has to rely only on the first transition. In any way, when analyzing the single isotopologues alone by LC‐MS/MS, no signals in the MRMs of the other isotopologues were visible, thus proving the absence of any cross‐talk in the LC‐MS/MS method. The method validation data given in Table 4 confirmed the accuracy of the developed dual stable isotope method.

**TABLE 4:** Validation data for the plasma folate quantitation.

Parameter	5‐CH_3_‐H_4_PteGlu	^13^C‐[Ph_6_]‐5‐CH_3_‐H_4_PteGlu	^15^N_7_‐5‐CH_3_‐H_4_PteGlu
LOD [nmol/L]	0.86	0.40	
LOQ[nmol/L]	3.23	1.45	
Recovery [%]	112 ± 12.8 101 ± 0.57 97.7 ± 5.04	99.3 ± 6.27 99.2 ± 1.41 101 ± 1.63	
Precision [RSD%] • Inter‐injection (*n* = 15) • Intra‐day (*n* = 3) • Inter‐day (*n* = 3)	2.06 2.96 1.32	2.39 2.25 4.87	2.19 3.70 3.55

### 3.3 Method Validation

The method for quantifying plasma folates was based on our previous reports [24, 30] with the addition of the new 5‐CH_3_‐H_4_PteGlu isotopologues. As [^15^N_7_]‐5‐CH_3_‐H_4_PteGlu was not available as a pure reference compound, its quantitation was performed like the other isotopologues based on the IS [^13^C_5_]‐5‐CH_3_‐H_4_PteGlu using the same calibration function as that for the unlabeled isotopologue. This adoption is possible due to the isotopic purity of the [^15^N_7_]‐labeled folates and the similarity of the calibration functions of unlabeled isotopologue and the [^13^C‐Ph]_6_‐isotopologue. Moreover, due to the lack of the pure compound, only precision data could be measured for [^15^N_7_]‐5‐CH_3_‐H_4_PteGlu. The method validation for the other isotopologues performed in analogy to Striegel et al. [24] revealed excellent sensitivities with LODs and LOQs below 1 and 3.3 nmol/L, respectively. Recoveries were, with percentages between 97 and 112%, in a very good range. Inter‐injection, intra‐day, and inter‐day precisions were all below 5 % RSD and confirmed the excellent performance of the method (Table 4).

### 3.4 Human Intervention Study

Six volunteers participated on the human study and their names were pseudonymized by a random three‐letter code (KCP, SLH, TDY, OUD, WVY, and MZM). Sexes were equally distributed; their BMIs were in the range of 20.2 – 29.0 kg/m^2^ and their age between 22 and 26 y. The study followed a random cross‐over design with three study days and a time lag of 2 weeks between the study days. On the intervention days, the participants obtained either the ^15^N‐labeled yeast containing 400 µg ^15^N‐labeled folates, the reference dose of 400 µg [^13^C‐Ph]_6_‐(6S)‐5‐CH_3_‐H_4_PteGlu and the control food, which was essentially free from folates.

Almost all interventions with isotope labeled doses showed clear plasma signals for the 5‐CH_3_‐H_4_PteGlu isotopologue, which referred to the given labeled folate(s). Moreover, the monitored labeled 5‐CH_3_‐H_4_PteGlu in plasma returned almost completely to zero after 24 h, which enabled us to calculate the complete AUC with less than 20 % falling out of the measurement period. Additionally, the plasma curves revealing partly sharp peak maxima confirmed the need of measuring the plasma folates in short intervals and monitoring 24 h to measure the complete AUC.

The only exception was subject MZM, for whom no plasma signal after the dosing of the reference substance was visible. As the reference substance was taken in a small amount of 400 µg and the subject struggled with swallowing all the capsules it might have happened that the reference substance was not completely taken in by the subject. Anyway, this subject was no longer considered in the assessment due to the missing reference data.

**FIGURE 3:**
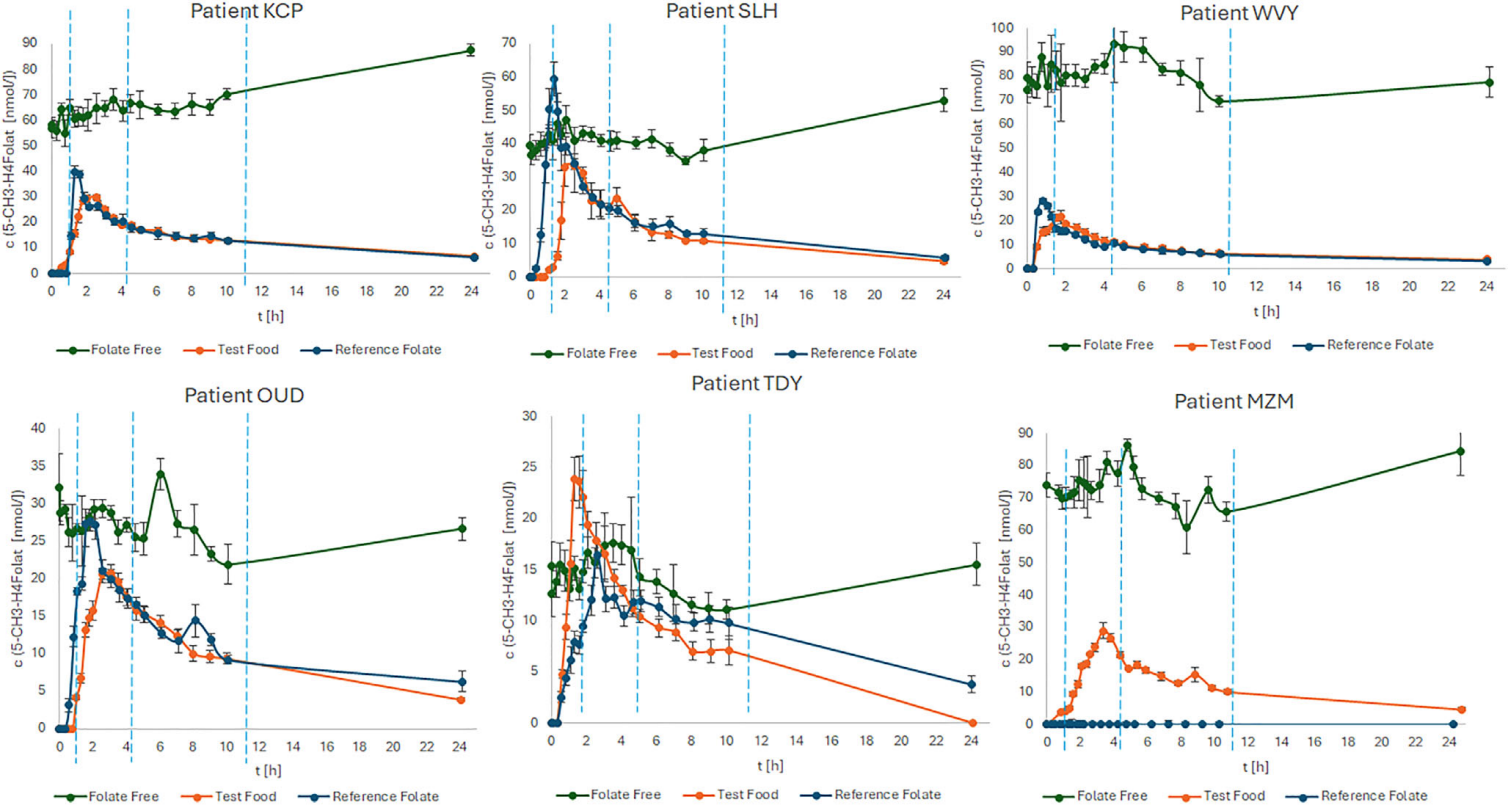
Mean plasma concentration [nmol L‐1] of the folate free day (green), the test day with the yeast (orange) and the reference day(blue) of all six subjects. For the folate free day, the plasma curve of unlabeled 5‐CH3‐H4PteGlu, for the test day with the yeast the curve of 15N7‐5‐CH3‐H4PteGlu and for the reference day the curve of 13C‐[Ph6]‐5‐CH3‐H4PteGlu is given, respectively. Standard deviation [nmol/L‐1], calculated from a technical triplicate.

Figure 3 shows the postabsorptive plasma concentrations of the folate‐free day, the test day with the labeled yeast, and the reference day with the labeled folate supplement for all subjects.

The plasma concentration curve of the folate‐free days showed no maximum for all individuals with average concentrations ranging between 15 nmol/L (subject TDY) and 85 nmol/L (subject WVY). These values demonstrate the high variations between the five individuals. These deviations do not only apply within different participants but also for the three different study days of the same participant. Especially, the highly variating start values c_0_ again highlight the problems if no labeled folate is used. For all biokinetic evaluations, only the respectively labeled 5‐CH_3_‐H_4_PteGlu plasma curves were used. The other folate vitamers (PteGlu, H_4_PteGlu, 5‐CHO‐H_4_PteGlu, and 10‐CHO‐PteGlu) were also monitored in all plasma samples, but the results were not considered in this study due to their low amounts under the estimated LoDs and LoQs.

Each dosage with a labeled folate revealed at least one clear maximum in the plasma curve, and in some of the curves two maxima could be observed. The time points of the peak maxima could be measured very accurately due to the short sampling intervals of every 15 min within the first 2 h after dosage. In mean the t_max_ after dosing the reference compound appeared at 1.6 h and was somewhat earlier than that (2.44 h) of the yeast tested. A second maximum was observed for the participants KCP (2.6 h), SLH (2.0 h), TDY (5.1 h), WVY (4.7 h) and OUD (8.1 h) for dosing the reference, whereas SLH (5.0 h) and MZM (5.4 h) showed the second maximum for dosing the yeast.

**FIGURE 4:**
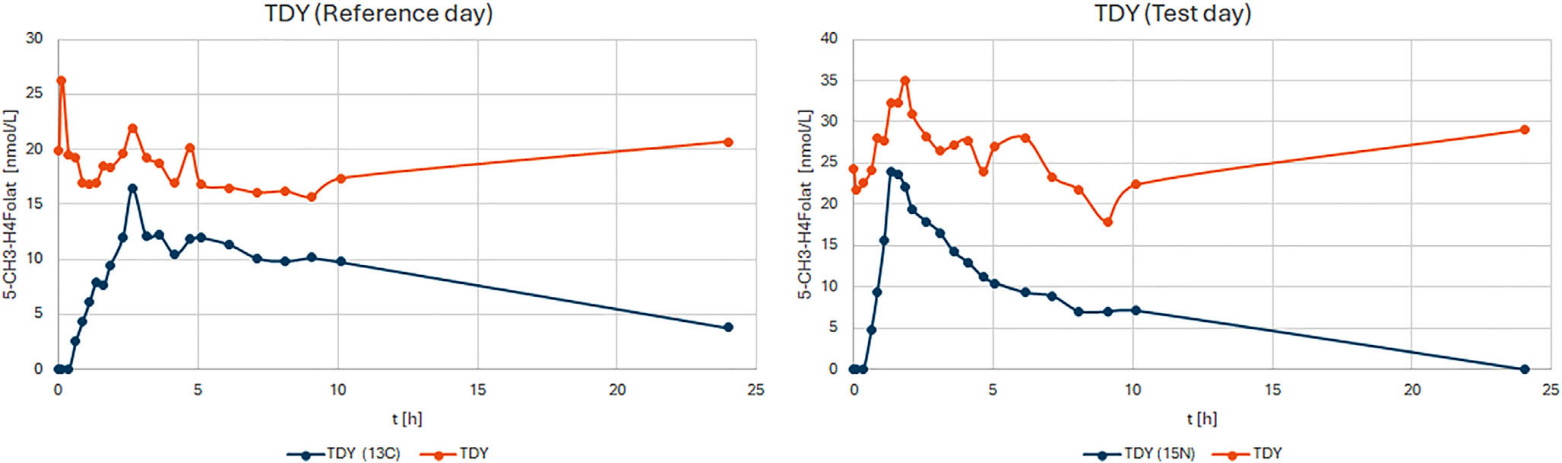
Mean plasma folate curve of labeled (blue) and unlabeled (orange) 5‐CH_3_‐H_4_PteGlu at the reference day and the test day of subject TDY illustrating the parallel measurement of the occurring isotopologues. Standard deviation [nmol/L^−1^], calculated from a technical triplicate.

During the reference day and the test day not only the labeled 5‐CH_3_‐H_4_PteGlu plasma curve was determined but also the unlabeled folate baseline of every patient. As representative example the folate plasma curve of patient TDY is shown in Figure 4.

The baseline folate levels revealed a mean 5‐CH_3_‐H_4_PteGlu concentration ranging from 25.5 nmol/L (SLH) to 53 nmol/L (KCP) during the yeast intervention, and from 18.6 nmol/L (TDY) to 59.2 nmol/L (KCP) during the reference intervention. Notably, the plasma curve of the unlabeled folate showed a maximum at the same timepoints as the respective labeled folate, although no unlabeled folates were administered. For all patients, folate baseline concentrations were consistently higher at each timepoint compared to the labeled curve. The only exception was Patient SLH, whose folate levels were either similar to or higher than the labeled curve at certain timepoints during both interventions.

Table 5 presents the biokinetic parameters of the three different study days.

**TABLE 5:** Biokinetic parameters for the three interventions of all included subjects (*n* = 5).

	Folate free control day	Yeast intervention (^15^N_7_‐5‐CH_3_‐H_4_PteGlu)	Reference intervention (^13^C[Ph]_6_‐5‐CH_3_‐H_4_PteGlu)
**c (1.detection)** ^a^ **[nmol/L]**	49.8 ± 25.0	4.42 ± 2.54	6.38 ± 9.71
**t (1. detection)** ^b^ **[h]**	0	0.81 ± 0.21	0.58 ± 0.18
**c_max_ [nmol/L]**	58.1 ± 30.3	26.4 ± 5.04	34.3 ± 16.2
**t_max_ [h]**	5.17 ± 2.72	2.44 ± 0.76	1.60 ± 0.69
**c_mean_ [nmol/L]**	50.4 ± 26.7	14.7 ± 2.07	6.9 ± 6.03
**Relative bioavailability [%] to 5‐CH_3_‐H_4_PteGlu**	**74 ± 11.0**		

^a^ concentration of first detected peak in plasma curve.

^b^ time of first detected peak in plasma curve.

At the plasma curve maxima, the reference compound showed a higher mean c_max_ of 36.0 nmol/L than that of the yeast with 29.1 nmol/L. With considering the slightly different doses, the mean AUC referred to a mean bioavailability of 74 % of the yeast folates relative to the reference compound. When comparing the calculated data for each subject, the bioavailabilities of the yeast folates ranged between 61% and 85 % (Table 5). Here it should be noted that the yeast folates consisted mainly of the hexa‐ and heptaglutamic forms, whereas the reference compound was a monoglutamate.

The calculation of the yeast folate bioavailabilities of the female and male subjects (Table 6) revealed means of 70 and 80 %, respectively. However, this difference was not significant. In this respect it has to be considered, that the one drop‐out subject was male thus resulting in only two data for the males compared to three data for the females.

**TABLE 6:** Biokinetic parameters.

	Subject SLH	Subject TDY	Subject KCP	Subject WVY	Subject OUD
**Sex [f/m]**	f			m	
**rel. bioavailability [%] to 5‐CH_3_‐H_4_PteGlu**	70 ± 13.0			80 ± 3.54	
**c_max_ (^15^N/^13^C)** ^a^ **[nmol/L]**	29.0 ± 4.72 / 38.5 ± 21.5			21.1 ± 0.66 / 28.0 ± 0.54	
**c_mean_(^15^N/^13^C)** ^b^ **[nmol/L]**	15.4 ± 2.34 / 18.3 ± 7.94			12.9 ± 0.32 / 14.8 ± 2.09	

^a^ maximum concentration of the respectively labeled 5‐CH_3_‐H_4_PteGlu in the plasma curve.

^b^ mean concentration of the respectively labeled 5‐CH_3_‐H_4_PteGlu in the plasma curve.

Another interesting aspect is a comparison of the kinetic data and bioavailabilities with respect to the genetic background of the subjects, although the yeast folates mainly consisted of 5‐CH_3_‐H_4_PteGlu vitamers and, therefore, a potentially limited conversion via MTHFR mutations should not affect the appearance of 5‐CH_3_‐H_4_PteGlu in plasma. In this study, we considered only the most relevant single nucleotide polymorphisms (SNPs) of the MTHFR gene with its C677C and A1298A polymorphisms. The respective biokinetic data are given in Table S9. In contrast to our expectations, the subjects with C677T and A1298A genotype revealed the highest bioavailabilities. However, due to the general variability of ± 10 % bioavailability and the small number of individuals for the single genotypes these differences cannot be considered significant.

## 4 Discussion

Extrinsically labelling of test foods for human studies reflects absorption from supplements or fortified foods, whereas intrinsically labeled foods are equivalent to usual foods with respect to the polyglutamate/monoglutamate spectrum, the different C1‐derivative distribution as well as folate incorporation in the matrix. Therefore, bioavailability can be determined considering all these effects in contrast to that of extrinsically labeled foods. There are only a few reports on the use of intrinsically labeled folates in foods. They mostly refer to spinach, which has been either labeled with ^13^C via pulse labelling with CO_2_ [40] or labeled with ^15^N by hydroponic cultivation and [^15^N]‐nitrate in the nutrient solution [41]. Moreover, spinach folates have also been labeled with ^14^C to be used in bioavailability studies applying acceleration mass spectrometry [42]. Yeast has also been labeled before with ^15^N like in the present study, but it has not been used to study folates, but for example, for investigating whole protein turnover [43] or quantitative proteomics [44]. An important prerequisite for using intrinsically labeled foods in bioavailability studies is a sufficient degree of labelling to enable unequivocal differentiation from the natural and other tracer isotopologues. The above‐mentioned intrinsically labeled spinach prepared from labeled CO_2_ revealed in its ^13^C version only a small isotopic enhancement of the m+2 *m/z* signal compared to its natural isotopologue [40]. However, feeding the spinach hydroponically with ^15^N resulted in the [^15^N_3_]‐isotopologue being the most abundant one, thus making this food feasible for a human bioavailability study [41]. This spinach was then successful in comparing the bioavailability of spinach folates with that of added 5‐CHO‐H_4_PteGlu and of PteGlu, as will be outlined below. The intrinsically labeled yeast produced in the present study, however, yielded an almost complete ^15^N labelling with the [^15^N_7_]‐isotopologue being almost the only one. Therefore, with respect to isotope labelling it was best suited to be used in our bioavailability study.

The applied tandem mass spectrometer was able to differentiate all four 5‐CH_3_‐H_4_PteGlu isotopologues, which would not have been possible with a single stage mass spectrometer. Up to present, only two different isotopologues have been applied in each of the various folate human studies, either [^2^H_2_]‐, [^2^H_2_]‐PteGlu_7_ and [^2^H_4_]‐PteGlu [45], or [^13^C_5_]‐ and [^2^H_2_]‐PteGlu [46], [^13^C‐Ph]_6_‐ and [^2^H_4_]‐PteGlu [47], [^13^C‐Ph]_6_‐PteGlu and [^13^C‐Ph]_6_‐5‐CHO‐H_4_PteGlu and [^15^N_1‐7_]‐folates [48].

Thus, we here introduce for the first time the design of dual isotopes and four isotopologues of a naturally occurring folate into folate research. For determining the AUC of the plasma response, the clear plasma 5‐CH_3_‐H_4_PteGlu curves offered the unique opportunity to accurately calculating AUC with a basal level of zero in contrast to previous studies, where either the unlabeled 5‐CH_3_‐H_4_PteGlu level at *t* = 0 had to be taken as base line or the minimum level throughout the monitored time period. Moreover, the end of the measurement interval was clear from the return of the 5‐CH_3_‐H_4_PteGlu levels to zero.

Our result of 74 % bioavailability of yeast folates relative to the reference 5‐CH_3_‐H_4_PteGlu adds further valuable insight into folate bioavailability. Previous short‐term studies compared the bioavailability of different folate vitamers, either different C1‐monoglutamates or monoglutamates and polyglutamates, or folates given orally as pure compounds or added to different food items or meals. It is generally well accepted that PteGlu has a different plasmakinetics to all other folates [48] and, therefore, is not appropriate as a reference dose in bioavailability studies. For this reason and as we did not use PteGlu in our present study, we will not discuss studies that used labeled PteGlu further. The incomplete bioavailability of yeast folates may be attributed essentially to three possible explanations: first, the yeast folates being almost all in their hepta‐ or hexaglutamate forms in contrast to the monoglutamate reference dose and, second, yeast folates being incorporated in the yeast cell matrix. The third possible explanation might be that the yeast folates are a mixture of different C1‐derivatives (5‐CH_3_‐H_4_PteGlu and H_4_PteGlu forms) with different properties, which also contrasts to the 5‐CH_3_‐H_4_PteGlu reference dose and may be an additional cause for the lower bioavailability.

For the latter explanation, the bioavailabilities of different C1‐derivatives, the literature is rather scarce. There have been only comparisons of natural folates to PteGlu, either 5‐CH_3_‐H_4_PteGlu or 5‐CHO‐H_4_PteGlu. Around 20 % of the folates of the yeast we used consisted of H_4_PteGlu and there is some evidence that the bioavailability of the latter might be compromised compared to 5‐CH_3_‐H_4_PteGlu due to the susceptibility of H_4_PteGlu with respect to oxidation. This is the rationale for the speculation of H_4_PteGlu being the cause for the low bioavailability of Camembert cheese folates, as the respective camembert rind showed a lower bioavailability [30, 50].

The different stabilities of the folate vitamers in general and during digestion may also be a reasoning for the differences in bioavailabilities. In this respect, the complete bioavailability and the stability of our reference compound 5‐CH_3_‐H_4_PteGlu has been questioned. When considering the general stability towards oxidation, the latter for sure is more susceptible than folic acid in general and possibly in food matrices. However in gastric in intestinal fluids, ascorbic acid prevents 5‐CH_3_‐H_4_PteGlu from major degradation [50], although wheat appears to adversely affect its stability during simulated digestion [51]. Moreover, comparisons of administration between 5‐methyltetrahydrofolate and folic acid clearly show higher plasma response of 5‐methyltetrahydrofolate [52] and thus, a potentially lower stability of 5‐CH_3_‐H_4_PteGlu shows no significant effect on bioavailability, at least in comparison to the “stable” compound folic acid and at least in the matrices investigated. In any case one still has to emphasize that the reported bioavailabilities are noabsolute values, but relative to our reference compound 5‐CH_3_‐H_4_PteGlu. Whether the absolute bioavailability of the latter is 100 % or less is still under debate as outlined in the introduction.

For the different bioavailabilities of poly and monoglutamates there have been several studies, but unfortunately, they are not conclusive. Whereas Wei et al. [45] showed for labeled PteGlu_6_ a lower bioavailability than PteGlu when added to orange juice, and Neuhouser et al. [53] reported a higher AUC for PteGlu relative to not further specified PteGlu polyglutamates in orange juice, different polyglutamate distributions in spinach [54] and in egg yolk, spinach and yeast [55] did not show an effect on folate bioavailability. For this reason, we cannot decisively attribute our result to the polyglutamates being prevalent in yeast.

The effect of the food matrix on folate bioavailability has been reported in many studies. However, almost all of them used extrinsically added PteGlu to the foods, for example, PteGlu added to bread or a breakfast [49].

The only study that is comparable with our present investigation is the bioavailability study of Wright et al. [48] using intrinsically labeled spinach. They compared the bioavailability of the intrinsically labeled folates with those of [^13^C‐Ph]_6_‐PteGlu and [^13^C‐Ph]_6_‐5‐CHO‐H_4_PteGlu. From the appearance of the respective 5‐CH_3_‐H_4_PteGlu in plasma the latter authors calculated an absolute bioavailability from the plasma concentrations and its kinetics. They found that the spinach folates show 221 % bioavailability and 5‐CHO‐H_4_PteGlu 180 % bioavailability relative to PteGlu. Apart from that in the latter study, intrinsically labeled folates have been used, the results are hardly comparable to ours though. We used 5‐CH_3_‐H_4_PteGlu as the reference dose in contrast to PteGlu and 5‐CHO‐H_4_PteGlu of the latter study. Moreover, the bioavailability of spinach folates cannot be referred to monoglutamates or polyglutamates as the respective spectrum has not been measured by Wright et al., and the spinach polyglutamate percentage of total folate may range between 0% and 60 % [55].

Concerning the generally assumed food folate bioavailability being around 50 %, our results with 74 % of yeast folates relative to 5‐CH_3_‐H_4_PteGlu are not too different, when considering that the food folates’ bioavailability in different foods may range in general between 10 % and 100%. In contrast to the long‐term study (30 days of intervention) by Hannon Fletcher et al. [29], who found a yeast bioavailability of 59% compared to folic acid, the present study observed a higher bioavailability of yeast folates. This discrepancy may be attributed to the variance in yeast folate contents, our limited number of participants, the short‐term design of our study, the use of plasma homcysteine as a biomarker in the latter study, the use of a different reference folate and the use of labeled folates as precise tracers in our study.

A further interesting aspect of our dual isotope study is the behavior of the plasma kinetics of unlabeled 5‐CH_3_‐H_4_PteGlu in general and after the administration of labeled folates. In general, the unlabeled 5‐CH_3_‐H_4_PteGlu level remained rather constant during the study days. However, and unexpectedly, after dosage of labeled folates the unlabeled curve showed similar peaks at the same time points as those of the labeled 5‐CH_3_‐H_4_PteGlu isotopologues (Figure 4). This effect can be explained with the assumption that the labeled folate enters into an isotope equilibrium with its unlabeled analogue when passing folate pools (e.g., mucosa or liver cells) and thus carrying the unlabeled folates into circulation. This may lead to a dilution of the labeled folate response in plasma, but this does not change the outcome of the relative bioavailability data as it affects the labeled yeast folates and the labeled reference folates likewise.

## 5 Conclusion

Our dual isotope design using four isotopologues was the first assay to study the bioavailabilities of endogenously occurring folates in encapsulated yeast as a model for food. Our biokinetic calculations were not restricted by fluctuations of the natural folate plasma level and reached a novel level of accuracy. Moreover, the rather homogenous batch of subjects decreased the fluctuations in individual variations and revealed a rather precise result, although only 5 subjects were evaluated. However, we have to admit several restrictions due to the pilot character of our trial:

First, the resulting bioavailability is only valid for yeast, which is not a common food and, up to present, is only partly used as food ingredient. Moreover, the yeast being lyophilized and dosed in a capsule is just a simplified model for a food. Therefore, the study would have to be extended to other foods with the limitation of generating intrinsically labeled study foods.

Second, our result is limited to the investigated small study cohort, which is not representative of the general population, Therefore, in further studies, validation in a larger group of volunteers must be conducted to obtain a more comprehensive result.

Third, with the prevalent folate distribution and the reference folate we were not able to rule out the decisive dependencies on folate bioavailability. Therefore, in further studies, other intrinsically labeled foods with different polyglutamate distributions and different C1‐derivatives would be needed, along with a better suited reference compound, for example, a 5‐CH_3_‐H_4_PteGlu polyglutamate. The overall goal of upcoming studies should be, on the one hand, assessing accurately the folate bioavailability of further and more relevant foods and, on the other hand, gaining more insight on the dependencies on folate bioavailability to be able to predict it from currently available analytical results as we already discussed earlier [50].

## Funding

The authors have nothing to report.

## Conflicts of Interest

The authors declare no conflicts of interest.

## Supporting information




**Supporting File**: mnfr70453‐sup‐0001‐SuppMat.docx.

## Data Availability

Data supporting this article have been included as part of the ESI. Further information will be made available upon request from the corresponding author.
