# Supplementary material for: Determination of Folate Bioavailability From Brewer's Yeast in a Dual Isotope Randomized Human Intervention Study
Source: Mol Nutr Food Res. 2026 Apr 9;70:e70453. doi: 10.1002/mnfr.70453 (PMC13063356; doi:10.1002/mnfr.70453)
Supplement: Supplementary file 1 — Supporting File: mnfr70453‐sup‐0001‐SuppMat.docx. [file MNFR-70-e70453-s001.docx]

**Supplementary Electronic Material**

### **Determination of folate bioavailability from brewer’s yeast in a dual isotope randomized human intervention study**

Nadine Weber, Lisa Striegel, Yvonne Methner, Beate Brandl, Viola Groehn, Jean-Pierre Knapp, Thomas Skurk, Michael Rychlik

**Supplementary Table S1**: Composition of the basic medium for cultivation of yeast for the human study.

| Compound for non-labelled medium / compound for isotope labelled medium; sources in superscript |  |
| --- | --- |
| Part A | Basic Medium |
| Ammonium chloride ^(1)^ / [^15^N] Ammonium chloride (≥ 98.0 atom % ^15^N, ≥ 99.0 %, (CP)) ^(2)^) ( | 5.00 g/L |
| Boric acid ^(1)^/ - | 5.00·10^-4^ g/L |
| Calcium chloride (2·H_2_O) ^(1)^ / Calcium chloride (2·H_2_O) (BioReagent, ≥ 99.0 %) ^(2)^ | 0.13  g/L |
| Iron sulphate (7·H_2_O) ^(1)^/ Iron sulphate (7·H_2_O) (BioReagent, ≥ 99.0 %) ^(2)^ | 2.00·10^-4^ g/L |
| Glucose ^(1)^/ Glucose (BioReagent, ≥ 99.5 %) ^(2)^ | 10.0 g/L |
| Potassium dihydrogenphosphate ^(1)^/ Potassium dihydrogenphosphate (BioReagent, ≥ 99.0 %) ^(2)^ | 1.00 g/L |
| Potassium iodide ^(3)^/ Potassium iodide (EMSURE®, ISO, Reag. Ph Eur)^(1)^ | 1.00·10^-4^ g/L |
| Copper sulphate (5·H_2_O) ^(1)^/ Copper sulphate (5·H_2_O) (EMSURE®, ACS, ISO, Reag. Ph Eur) ^(1)^ | 4.00·10^-5^ g/L |
| Magnesium sulphate (7·H_2_O) ^(3)^/ Magnesium sulphate (7·H_2_O) (BioReagent, ≥ 99.0 %) ^(2)^ | 1.00 g/L |
| Manganese sulphate ^(1)^/ Manganese-(II)-sulfat (1·H_2_O) (EMSURE®, ACS, Reag. Ph Eur) ^(1)^ | 4.00·10^-4^ g/L |
| Sodium chloride ^(3)^/ Sodium chloride (BioReagent, ≥ 99.0 %) ^(2)^ | 1.00·10^-1^ g/L |
| Sodium molybdate ^(4)^/ Sodium molybdate (2·H_2_O) (≥ 99.5 %) ^(2)^ | 2.00·10^-4^ g/L |
| Oleic acid ^(4)^/ Oleic acid (BioReagent) ^(2)^ | 0.1 ml/L |
| Zinc sulphate (1·H_2_O)^(2)^/ Zinc sulphate (7·H_2_O) ( Puriss. P.a., ACS reagent, reag. ISO. Reag. Ph. Eur., ≥ 99.5 %) ^(2)^ | 4.4·10^-4^ g/L /6.9·10^-4^ g/L |
|  |  |
| Part B (source) | Basic medium |
| Myo-Inositol ^(2)^/ Myo-Inositol (BioReagent) ^(2)^ | 2.00·10^-3^ g/L |
| d-Biotin^(5)^ / d-Biotin ( BioReagent, ≥ 99.0 %)^(2)^ | 2.00 µg/L |
| Calcium D-pantothenate ^(2)^/ Calcium D-pantothenate (BioReagent) ^(2)^ | 400 µg/L |
| Nicotinic acid ^(5)^/ Nicotinic acid (BioReagent, ≥ 98.0 %)^(2)^ | 400 µg/L |
| Pyridoxine hydrochloride ^(5)^/ Pyridoxine hydrochloride (BioReagent)^(2)^ | 400 µg/L |
| Riboflavin ^(5)^ / Riboflavin (BioReagent, ≥ 98.0 %)^(2)^ | 200 µg/L |
| Thiamine ^(5)^ / Thiamine (BioReagent)^(2)^ | 400 µg/L |

^1^ Merck KGaA, Darmstadt, Germany; ^2^ Sigma-Aldrich, Steinheim, Germany; ^3^ VWR International GmbH, Darmstadt, Germany; ^4^ Fluka Analytical/ Sigma-Aldrich, Steinheim, Germany; ^5^ Supelco, Bellefonte, USA

**Supplementary Table S2**: Total folate content of consumed food during the study.

| Food | Total folate [µg/100g] ± RSD [%] | Time of consumption |
| --- | --- | --- |
| Rice | **42.9** ± 3.06 | **Consumed only on the evening before study day** |
| Rice waffles | **46.4** ± 11.8 | **Consumed during study day in an unlimited amount during specified timepoints** |
| Maize waffles | **30.9** ± 4.51 |  |
| Cheese (1)-  Gouda | **24.2** ± 3.22 |  |
| Cheese (2)-Emmentaler | **13.6** ± 90.9 |  |
| Cherry Marmalade | **3.77** ± 18.4 |  |
| Honey | **2.52** ± 80.6 |  |
| Cellulose | **5.50** ± 23.6 |  |
| Capsule | **0.00** |  |

All consumed food was weighed and recorded in a diary by the patient.

RSD: relative standard deviation

**Supplementary Table S3**: MRM scan parameters for the folate vitamers and their isotopologues in blood plasma samples; detection in positive ESI mode.

| Compound | Precursor ion  *m/z* | Product ion *m/z* | Dwell Time (msec) | Q1 Pre Bias (V) | CE | Q3 Pre Bias (V) |
| --- | --- | --- | --- | --- | --- | --- |
| H_4_folate | 446.00  446.00  446.00 | 299.20  120.10  166.15 | 52.0  52.0  52.0 | -22.0  -22.0  -22.0 | -20.0  -37.0  -41.0 | -16.0  -14.0  -19.0 |
| [^15^N_7_]-H_4_folate | 453.00  453.00  453.00 | 305.20  121.10  171.15 | 52.0  52.0  52.0 | -22.0  -22.0  -22.0 | -20.0  -37.0  -41.0 | -16.0  -14.0  -19.0 |
| 5-CH_3_-H_4_folate | 460.20  460.20  460.20 | 313.20  180.15  194.25 | 38.0  38.0  38.0 | -13.0  -13.0  -23.0 | -20.0  -37.0  -33.0 | -17.0  -14.0  -22.0 |
| [^13^C_5_]-5-CH_3_-H_4_folate  (5-CH_3_-H_4_Pte[^13^C_5_]Glu) | 465.30  465.30  465.30 | 313.20  180.15  194.25 | 38.0  38.0  38.0 | -13.0  -13.0  -23.0 | -20.0  -37.0  -33.0 | -17.0  -14.0  -22.0 |
| [^15^N_7_]-5-CH_3_-H_4_folate | 467.20  467.20  467.20 | 319.20  185.15  199.25 | 38.0  38.0  38.0 | -13.0  -13.0  -23.0 | -20.0  -37.0  -33.0 | -17.0  -14.0  -22.0 |
| [^13^C-Ph]_6_-5-CH_3_-H_4_folate | 466.20  466.20  466.20 | 319.20  180.10  194.25 | 38.0  38.0  38.0 | -25.0  -18.0  -23.0 | -19.0  -40.0  -33.0 | -16.0  -20.0  -22.0 |
| 5-CHO-H_4_folate | 474.30  474.30  474.30 | 327.15  299.20  208.20 | 38.0  38.0  38.0 | -14.0  -14.0  -18.0 | -20.0  -31.0  -36.0 | -17.0  -16.0  -24.0 |
| [^15^N_7_]-5-CHO-H_4_folate | 481.30  481.30  481.30 | 333.15  305.20  213.20 | 38.0  38.0  38.0 | -14.0  -14.0  -18.0 | -20.0  -31.0  -36.0 | -17.0  -16.0  -24.0 |
| 10-CHO-PteGlu | 470.10  470.10  470.10 | 295.10  176.10  120.10 | 38.0  38.0  38.0 | -14.0  -24.0  -14.0 | -25.0  -42.0  -40.0 | -22.0  -20.0  -22.0 |
| [^15^N_7_]-10-CHO-PteGlu | 477.10  477.10  477.10 | 301.10  181.10  121.10 | 38.0  38.0  38.0 | -14.0  -24.0  -14.0 | -25.0  -42.0  -40.0 | -22.0  -20.0  -22.0 |
| PteGlu | 442.30  442.30  442.30 | 295.15  176.20  120.05 | 52.0  52.0  52.0 | -13.0  -13.0  -13.0 | -16.0  -37.0  -35.0 | -16.0  -20.0  -14.0 |
| [^15^N_7_]-PteGlu | 449.30  449.30  449.30 | 301.15  181.20  121.05 | 52.0  52.0  52.0 | -13.0  -13.0  -13.0 | -16.0  -37.0  -35.0 | -16.0  -20.0  -14.0 |

**Supplementary Table S4**: MRM scan parameters for the folate monoglutamates and their isotopologues in food samples; detection in positive ESI mode.

| Compound | Precursor ion  *m/z* | Product ion *m/z* | Dwell Time (msec) | Q1 Pre Bias (V) | CE | Q3 Pre Bias (V) |
| --- | --- | --- | --- | --- | --- | --- |
| H_4_folate | 446.15  446.15  446.15 | 299.25  120.20  166.20 | 107.0  107.0  107.0 | -24.0  -24.0  -24.0 | -20.0  -38.0  -45.0 | -22.0  -24.0  -18.0 |
| [^13^C_5_]-H_4_folate (H_4_Pte[^13^C_5_]Glu) | 451.20  451.20  451.20 | 299.25  120.20  166.20 | 107.0  107.0  107.0 | -22.0  -16.0  -16.0 | -16.0  -38.0  -41.0 | -22.0  -14.0  -18.0 |
| [^15^N_7_]-H_4_folate | 453.00  453.00  453.00 | 305.20  121.10  171.15 | 107.0  107.0  107.0 | -24.0  -24.0  -24.0 | -20.0  -38.0  -45.0 | -22.0  -24.0  -18.0 |
| 5-CH_3_-H_4_folate | 460.10  460.10  460.10 | 313.30  180.20  194.20 | 67.0  67.0  67.0 | -26.0  -24.0  -14.0 | -19.0  -20.0  -35.0 | -16.0  -30.0  -20.0 |
| [^13^C_5_]-5-CH_3_-H_4_folate  (5-CH_3_-H_4_Pte[^13^C_5_]Glu) | 465.20  465.20  465.20 | 313.25  180.20  194.25 | 67.0  67.0  67.0 | -24.0  -24.0  -24.0 | -20.0  -35.0  -30.0 | -16.0  -20.0  -14.0 |
| [^15^N_7_]-5-CH_3_-H_4_folate | 467.20  467.20  467.20 | 319.20  185.15  199.25 | 67.0  67.0  67.0 | -26.0  -24.0  -14.0 | -19.0  -20.0  -35.0 | -16.0  -30.0  -20.0 |
| 5-CHO-H_4_folate | 474.10  474.10  474.10 | 327.20  299.20  166.25 | 67.0  67.0  67.0 | -14.0  -26.0  -14.0 | -20.0  -32.0  -44.0 | -16.0  -22.0  -12.0 |
| [^13^C_5_]-5-CHO-H_4_folate  (5-CHO-H_4_Pte[^13^C_5_]Glu) | 479.15  479.15  479.15 | 327.20  299.20  166.30 | 67.0  67.0  67.0 | -14.0  -24.0  -14.0 | -20.0  -32.0  -48.0 | -24.0  -42.0  -18.0 |
| [^15^N_7_]-5-CHO-H_4_folate | 481.30  481.30  481.30 | 333.15  305.20  213.20 | 67.0  67.0  67.0 | -14.0  -26.0  -14.0 | -20.0  -32.0  -48.0 | -16.0  -22.0  -12.0 |
| 10-CHO-PteGlu | 470.10  470.10  470.10 | 295.20  176.20  120.20 | 49.0  49.0  49.0 | -24.0  -24.0  -14.0 | -26.0  -41.0  -40.0 | -20.0  -20.0  -14.0 |
| [^13^C_5_]-10-CHO-PteGlu  (10-CHO-Pte[^13^C_5_]Glu) | 475.15  475.15  475.15 | 295.15  176.20  120.10 | 49.0  49.0  49.0 | -24.0  -24.0  -24.0 | -24.0  -40.0  -41.0 | -22.0  -28.0  -20.0 |
| [^15^N_7_]-10-CHO-PteGlu | 477.10  477.10  477.10 | 301.10  181.10  121.10 | 49.0  49.0  49.0 | -24.0  -24.0  -14.0 | -26.0  -41.0  -40.0 | -20.0  -20.0  -14.0 |
| PteGlu | 442.10  442.10  442.10 | 176.15  295.20  120.15 | 67.0  67.0  67.0 | -24.0  -22.0  -24.0 | -39.0  -20.0  -34.0 | -18.0  -22.0  -28.0 |
| Pte[^13^C_5_]Glu | 447.10  447.10  447.10 | 295.20  176.25  120.20 | 67.0  67.0  67.0 | -24.0  -22.0  -24.0 | -18.0  -38.0  -45.0 | -22.0  -12.0  -12.0 |
| [^15^N_7_]-PteGlu | 449.30  449.30  449.30 | 301.15  181.20  121.05 | 67.067.067.0 | -24.0  -22.0  -24.0 | -39.0-20.0-34.0 | -18.0  -22.0  -28.0 |

Gradient elution for LC-MS/MS of the folate monoglutamates and their isotopologues in food samples:

0.10 Autosampler Inject

1.00 Pumps Pump B Conc. 3

3.00 Pumps Pump B Conc. 10

5.50 Pumps Pump B Conc. 10

10.50 Pumps Pump B Conc. 15

11.50 Pumps Pump B Conc. 50

12.50 Pumps Pump B Conc. 50

13.50 Pumps Pump B Conc. 5

17.50 Pumps Pump B Conc. 5

17.50 Controller Stop

**Supplementary Table S5**: MRM scan parameters for the folate polyglutamates and their isotopologues in food samples; detection in positive ESI mode.

| Compound | Precursor ion  *m/z* | Product ion *m/z* | Dwell Time (msec) | Q1 Pre Bias (V) | CE | Q3 Pre Bias (V) |
| --- | --- | --- | --- | --- | --- | --- |
| H_4_folate  (H_4_PteGlu_1_) | 446.00 | 299.20 | 5.0 | -22.0 | -20.0 | -16.0 |
|  | 446.00 | 120.10 | 5.0 | -22.0 | -37.0 | -14.0 |
|  | 446.00 | 166.15 | 5.0 | -22.0 | -41.0 | -19.0 |
| H_4_PteGlu_2_ | 575.30 | 299.10 | 10.0 | -28.0 | -35.0 | -28.0 |
|  | 575.30 | 166.10 | 10.0 | -38.0 | -61.0 | -28.0 |
| H_4_PteGlu_3_ | 352.65 | 299.10 | 10.0 | -20.0 | -15.0 | -22.0 |
|  | 352.65 | 166.10 | 10.0 | -20.0 | -33.0 | -20.0 |
| H_4_PteGlu_4_ | 417.15 | 299.10 | 10.0 | -12.0 | -16.0 | -21.0 |
|  | 417.15 | 166.10 | 10.0 | -12.0 | -36.0 | -13.0 |
| H_4_PteGlu_5_ | 481.65 | 299.10 | 10.0 | -28.0 | -15.0 | -34.0 |
|  | 481.65 | 166.10 | 10.0 | -28.0 | -39.0 | -20.0 |
| H_4_PteGlu_6_ | 546.15 | 299.10 | 10.0 | -18.0 | -20.0 | -34.0 |
|  | 546.15 | 166.10 | 10.0 | -18.0 | -45.0 | -20.0 |
| H_4_PteGlu_7_ | 610.65 | 299.10 | 10.0 | -34.0 | -20.0 | -36.0 |
|  | 610.65 | 166.10 | 10.0 | -34.0 | -53.0 | -20.0 |
| 5-CH_3_-H_4_folate | 460.20 | 313.20 | 6.0 | -13.0 | -20.0 | -17.0 |
|  | 460.20 | 180.15 | 6.0 | -13.0 | -37.0 | -14.0 |
|  | 460.20 | 194.25 | 6.0 | -23.0 | -33.0 | -22.0 |
| [^13^C_5_]-5-CH_3_-H_4_folate | 465.30 | 313.20 | 6.0 | -13.0 | -20.0 | -17.0 |
|  | 465.30 | 180.15 | 6.0 | -13.0 | -37.0 | -14.0 |
|  | 465.30 | 194.25 | 6.0 | -23.0 | -33.0 | -22.0 |
| 5-CH_3_-H_4_PteGlu_2_ | 589.30 | 313.20 | 11.0 | -28.0 | -25.0 | -25.0 |
|  | 589.30 | 180.15 | 11.0 | -36.0 | -62.0 | -25.0 |
| 5-CH_3_-H_4_PteGlu_3_ | 359.65 | 313.20 | 10.0 | -20.0 | -15.0 | -22.0 |
|  | 359.65 | 180.15 | 10.0 | -20.0 | -33.0 | -20.0 |
| 5-CH_3_-H_4_PteGlu_4_ | 424.15 | 313.20 | 10.0 | -12.0 | -16.0 | -21.0 |
|  | 424.15 | 180.15 | 10.0 | -12.0 | -36.0 | -13.0 |
| 5-CH_3_-H_4_PteGlu_5_ | 488.65 | 313.20 | 10.0 | -28.0 | -15.0 | -34.0 |
|  | 488.65 | 180.15 | 10.0 | -28.0 | -39.0 | -20.0 |
| 5-CH_3_-H_4_PteGlu_6_ | 553.20 | 313.20 | 10.0 | -18.0 | -20.0 | -34.0 |
|  | 553.20 | 180.15 | 10.0 | -18.0 | -45.0 | -20.0 |
| 5-CH_3_-H_4_PteGlu_7_ | 617.70 | 313.20 | 10.0 | -34.0 | -20.0 | -36.0 |
|  | 617.70 | 180.15 | 10.0 | -34.0 | -53.0 | -20.0 |
| 5-CH_3_-H_4_PteGlu_8_ | 682.20 | 313.20 | 10.0 | -12.0 | -16.0 | -21.0 |
|  | 682.20 | 180.15 | 10.0 | -12.0 | -36.0 | -13.0 |
| 5-CHO-H_4_folate | 474.30 | 327.15 | 5.0 | -14.0 | -20.0 | -17.0 |
|  | 474.30 | 299.20 | 5.0 | -14.0 | -31.0 | -16.0 |
|  | 474.30 | 208.20 | 5.0 | -18.0 | -36.0 | -24.0 |
| 5- CHO -H_4_PteGlu_2_ | 603.20 | 327.10 | 5.0 | -28.0 | -25.0 | -25.0 |
|  | 603.20 | 299.10 | 5.0 | -36.0 | -65.0 | -25.0 |
|  | 603.20 | 208.20 | 5.0 | -36.0 | -65.0 | -25.0 |
| 5- CHO -H_4_PteGlu_3_ | 366.60 | 327.10 | 10.0 | -20.0 | -15.0 | -22.0 |
|  | 366.60 | 299.10 | 10.0 | -20.0 | -33.0 | -20.0 |
| 5- CHO -H_4_PteGlu_4_ | 431.10 | 327.10 | 10.0 | -12.0 | -16.0 | -21.0 |
|  | 431.10 | 299.10 | 10.0 | -12.0 | -36.0 | -13.0 |
| 5- CHO -H_4_PteGlu_5_ | 495.60 | 327.10 | 10.0 | -28.0 | -15.0 | -34.0 |
|  | 495.60 | 299.10 | 10.0 | -28.0 | -39.0 | -20.0 |
| 5- CHO -H_4_PteGlu_6_ | 560.10 | 327.10 | 10.0 | -18.0 | -20.0 | -34.0 |
|  | 560.10 | 299.10 | 10.0 | -18.0 | -45.0 | -20.0 |
| 5- CHO -H_4_PteGlu_7_ | 624.60 | 327.10 | 10.0 | -34.0 | -20.0 | -36.0 |
|  | 624.60 | 299.10 | 10.0 | -34.0 | -53.0 | -20.0 |
| 10-CHO-PteGlu_1_ | 470.00 | 295.15 | 5.0 | -23.0 | -26.0 | -22.0 |
|  | 470.00 | 176.20 | 5.0 | -23.0 | -40.0 | -20.0 |
|  | 470.00 | 120.15 | 5.0 | -17.0 | -39.0 | -14.0 |
| 10-CHO-PteGlu_2_ | 599.20 | 295.00 | 10.0 | -28.0 | -35.0 | -28.0 |
|  | 599.20 | 176.10 | 10.0 | -38.0 | -61.0 | -28.0 |
| 10-CHO-PteGlu_3_ | 364.60 | 295.00 | 10.0 | -20.0 | -15.0 | -22.0 |
|  | 364.60 | 176.10 | 10.0 | -20.0 | -33.0 | -20.0 |
| 10-CHO-PteGlu_4_ | 429.10 | 295.00 | 10.0 | -12.0 | -16.0 | -21.0 |
|  | 429.10 | 176.10 | 10.0 | -12.0 | -36.0 | -13.0 |
| 10-CHO-PteGlu_5_ | 493.60 | 295.00 | 10.0 | -28.0 | -15.0 | -34.0 |
|  | 493.60 | 176.10 | 10.0 | -28.0 | -39.0 | -20.0 |
| 10-CHO-PteGlu_6_ | 558.10 | 295.00 | 10.0 | -18.0 | -20.0 | -34.0 |
|  | 558.10 | 176.10 | 10.0 | -18.0 | -45.0 | -20.0 |
| 10-CHO-PteGlu_7_ | 622.60 | 295.00 | 10.0 | -34.0 | -20.0 | -36.0 |
|  | 622.60 | 176.10 | 10.0 | -34.0 | -53.0 | -20.0 |
| PteGlu_1_ | 442.10 | 176.15 | 10.0 | 34.0 | -24.0 | -39.0 |
|  | 442.10 | 295.20 | 10.0 | 34.0 | -22.0 | -20.0 |
|  | 442.10 | 120.15 | 10.0 | 34.0 | -24.0 | -34.0 |
| PteGlu_2_ | 571.20 | 295.10 | 10.0 | -28.0 | -35.0 | -28.0 |
|  | 571.20 | 176.30 | 10.0 | -38.0 | -61.0 | -28.0 |
| PteGlu_3_ | 350.60 | 295.10 | 10.0 | -20.0 | -15.0 | -22.0 |
|  | 350.60 | 176.30 | 10.0 | -20.0 | -33.0 | -20.0 |
| PteGlu_4_ | 415.10 | 295.10 | 10.0 | -12.0 | -16.0 | -21.0 |
|  | 415.10 | 176.30 | 10.0 | -12.0 | -36.0 | -13.0 |
| PteGlu_5_ | 479.60 | 295.10 | 10.0 | -28.0 | -15.0 | -34.0 |
|  | 479.60 | 176.30 | 10.0 | -28.0 | -39.0 | -20.0 |
| PteGlu_6_ | 544.10 | 295.10 | 10.0 | -18.0 | -20.0 | -34.0 |
|  | 544.10 | 176.30 | 10.0 | -18.0 | -45.0 | -20.0 |
| PteGlu_7_ | 608.60 | 295.10 | 10.0 | -34.0 | -20.0 | -36.0 |
|  | 608.60 | 176.30 | 10.0 | -34.0 | -53.0 | -20.0 |

**Supplementary Table S6**: MRM scan parameters for the [^15^N_7_]-5-CH_3_-H_4_folate polyglutamates in the yeast sample; detection in positive ESI mode.

| Compound | Precursor ion  *m/z* | Product ion *m/z* | Dwell Time (msec) | Q1 Pre Bias (V) | CE | Q3 Pre Bias (V) |
| --- | --- | --- | --- | --- | --- | --- |
| 5-CH_3_-H_4_folate | 460.10  460.10  460.10 | 313.30  180.20  194.20 | 70.0  70.0  70.0 | -20.0  -37.0  -33.0 | -20.0  -37.0  -33.0 | -17.0  -14.0  -22.0 |
| [^15^N_7_]-5-CH_3_-H_4_folate | 467.20 | 319.20 | 70.0 | -13.0 | -20.0 | -17.0 |
|  | 467.20 | 185.15 | 70.0 | -13.0 | -37.0 | -14.0 |
|  | 467.20 | 199.25 | 70.0 | -23.0 | -33.0 | -22.0 |
| [^13^C_5_]-5-CH_3_-H_4_folate | 465.30 | 313.20 | 70.0 | -13.0 | -20.0 | -17.0 |
|  | 465.30 | 180.15 | 70.0 | -13.0 | -37.0 | -14.0 |
|  | 465.30 | 194.25 | 70.0 | -23.0 | -33.0 | -22.0 |
| [^15^N_7_]-5-CH_3_-H_4_PteGlu_2_ | 597.30 | 319.20 | 70.0 | -28.0 | -35.0 | -28.0 |
|  | 597.30 | 185.15 | 70.0 | -38.0 | -61.0 | -28.0 |
| [^15^N_7_]-5-CH_3_-H_4_PteGlu_3_ | 364.15 | 319.20 | 70.0 | -20.0 | -15.0 | -22.0 |
|  | 364.15 | 185.15 | 70.0 | -20.0 | -33.0 | -20.0 |
| [^15^N_7_]-5-CH_3_-H_4_PteGlu_4_ | 429.15 | 319.20 | 70.0 | -12.0 | -16.0 | -21.0 |
|  | 429.15 | 185.15 | 70.0 | -12.0 | -36.0 | -13.0 |
| [^15^N_7_]-5-CH_3_-H_4_PteGlu_5_ | 494.15 | 319.20 | 70.0 | -28.0 | -15.0 | -34.0 |
|  | 494.15 | 185.15 | 70.0 | -28.0 | -39.0 | -20.0 |
| [^15^N_7_]-5-CH_3_-H_4_PteGlu_6_ | 559.20 | 319.20 | 70.0 | -18.0 | -20.0 | -34.0 |
|  | 559.20 | 185.15 | 70.0 | -18.0 | -45.0 | -20.0 |
| [^15^N_7_]-5-CH_3_-H_4_PteGlu_7_ | 624.20 | 319.20 | 70.0 | -34.0 | -20.0 | -36.0 |
|  | 624.20 | 185.15 | 70.0 | -34.0 | -53.0 | -20.0 |

**Supplementary Table S7**: Crossover design of the intervention study

| *Subject* | *Intervention Day 1* | *Intervention Day 2* | *Intervention Day 3* |
| --- | --- | --- | --- |
| MZM | 1 | 3 | 2 |
| WVY | 1 | 3 | 2 |
| SLH | 3 | 1 | 2 |
| TDY | 1 | 3 | 2 |
| OUD | 3 | 2 | 1 |
| KCP | 3 | 1 | 2 |

Test foods: 1: folate free, 2: ^15^N-labelled yeast, 3: Reference folate

**Supplementary Table S8:** Screening results of the participants of the human study.

| Participant | **KCP** | **OUD** | **TDY** | **SLH** | **WVY** | **MZM** | **Ø** |
| --- | --- | --- | --- | --- | --- | --- | --- |
| **Age [years]** | 25 | 26 | 23 | 24 | 22 | 24 | **24 ± 1.41** |
| **Sex [f/m]** | f | m | f | f | m | m |  |
| **BMI [kg/m^2]** | 20.2 | 29.0 | 22.0 | 27.8 | 25.5 | 21.5 | **24.0 ± 3.63** |
| **MTHFR genotypes** | C677T | C677T | C677C | C677C | C677T | C677T |  |
|  | A1298A | A1298A | A1298C | A1298C | A1298C | A1298A |  |
| **Vitamin B12 [pg/mL] (ref: 246-911 pg/mL)** | 319 | 372 | 371 | 282 | 371 | 256 | **329 ± 51.0** |
| **Homocysteine [µmol/ml] (ref: 5-12 µmol/mL)** | 13.2 | 13.7 | 12.7 | 12.7 | 10.7 | 13.1 | **13 ± 1.04** |
| **Hemoglobin [g/dL] (ref: f:12-16 g/dL; m:13.5-17.5 g/dL)** | 13.7 | 14.7 | 15.8 | 12.4 | 15 | 15.3 | **14 ± 1.24** |

Ref: reference

**Supplementary Table S9**  Biokinetic parameters of the most relevant single nucleotide polymorphisms (SNPs) of the MTHFR gene with its C677C and A1298A polymorphisms. The subjects with C677T and A1298A genotype revealed the highest bioavailabilities of > 83 %, whereas the volunteer with C677T and A1298C revealed 78 % bioavailability and the two subjects with C677C and A1298C genotype had the lowest bioavailability < 64 %.

| Participant | **SLH** | **TDY** | **WVY** | **OUD** | **KCP** |
| --- | --- | --- | --- | --- | --- |
| **MTHFR-genotypes** | C677C | C677C | C677T | C677T | C677T |
|  | A1298C | A1298C | A1298C | A1298A | A1298A |
| **rel. bioavailability [%]to 5-CH_3_-H_4_PteGlu** | 61 | 64 | 78 | 83 | 85 |
| **t (1.detection)^a^ (^15^N/^13^C) [h]** | 0.8 ± 0.32/  0.50 ± 0.19 | | 0.53/ 0.55 | 0.90 ± 0.18/  0.70 ± 0.19 | |
| **c_max_ (^15^N/^13^C)**  **[nmol/L]** | 28.6 ± 6.60 /  37.9 ± 30.3 | | 21.6 /  24.1 | 25.2 ± 6.48 /  33.6 ± 8.59 | |
| **t_max_ (^15^N/^13^C) [h]** | 1.90 ± 0.88/  2.0 ± 0.88 | | 1.80/0.80 | 2.8 ±0.35 /  1.60 ± 0.37 | |
| **c_mean_(^15^N/^13^C)**  **[nmol/L]** | 14.7 ± 2.84 /  17.8 ± 11.1 | | 12.7 /  13.3 | 14.9 ± 2.60 /  17.9 ± 2.28 | |

^a^ time of first detected peak in plasma curve

**
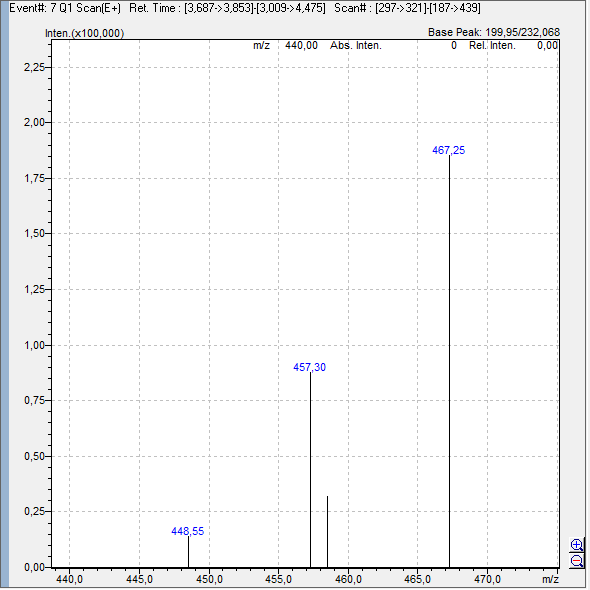
**

**Supplementary Figure S1**: LC-MS Spectrum of 5-CH3-H4PteGlu in intrinsically [15N]-labelled yeast showing the signal of the [15N7]-isotopologue at m/z 467
